# Pediatric emergency medicine point-of-care ultrasound: summary of the evidence

**DOI:** 10.1186/s13089-016-0049-5

**Published:** 2016-11-03

**Authors:** Jennifer R. Marin, Alyssa M. Abo, Alexander C. Arroyo, Stephanie J. Doniger, Jason W. Fischer, Rachel Rempell, Brandi Gary, James F. Holmes, David O. Kessler, Samuel H. F. Lam, Marla C. Levine, Jason A. Levy, Alice Murray, Lorraine Ng, Vicki E. Noble, Daniela Ramirez-Schrempp, David C. Riley, Turandot Saul, Vaishali Shah, Adam B. Sivitz, Ee Tein Tay, David Teng, Lindsey Chaudoin, James W. Tsung, Rebecca L. Vieira, Yaffa M. Vitberg, Resa E. Lewiss

**Affiliations:** 1 Children’s Hospital of Pittsburgh, 4401 Penn Ave, AOB Suite 2400, Pittsburgh, PA 15224 USA; 2 Children’s National Medical Center, Washington DC, USA; 3 Maimonides Medical Center, Brooklyn, NY USA; 4 New York Methodist Hospital, Brooklyn, NY USA; 5 Hospital for Sick Children, Toronto, Ontario Canada; 6 Boston Children’s Hospital, Boston, MA USA; 7 Queens Medical Center, Honolulu, HI USA; 8 University of California-Davis, Sacramento, CA USA; 9 Morgan Stanley Children’s Hospital, New York, NY USA; 10 University of California-San Diego, San Diego, CA USA; 11 Dell Children’s Medical Center, Austin, USA; 12 Boston Medical Center, Boston, MA USA; 13 Massachusetts General Hospital, Boston, MA USA; 14 Boston University Medical Center, Boston, NY USA; 15 Columbia University Medical Center, New York, NY USA; 16 St. Lukes-Roosevelt Hospital, New York, NY USA; 17 Montefiore Medical Center, Bronx, NY USA; 18 Newark Beth Israel Medical Center, Newark, NJ USA; 19 Mount Sinai Hospital, New York, NY USA; 20 Cohen Children’s Medical Center, New Hyde Park, USA; 21 Weill Cornell Medical Center, New York, NY USA; 22 University of Colorado, Aurora, CO USA

**Keywords:** Pediatric emergency medicine, Point-of-care ultrasound, Diagnostic, Procedures

## Abstract

The utility of point-of-care ultrasound is well supported by the medical literature. Consequently, pediatric emergency medicine providers have embraced this technology in everyday practice. Recently, the American Academy of Pediatrics published a policy statement endorsing the use of point-of-care ultrasound by pediatric emergency medicine providers.  To date, there is no standard guideline for the practice of point-of-care ultrasound for this specialty. This document serves as an initial step in the detailed “how to” and description of individual point-of-care ultrasound examinations.  Pediatric emergency medicine providers should refer to this paper as reference for published research, objectives for learners, and standardized reporting guidelines.

In the last 20 years “clinician-performed” or “point-of-care” ultrasound has expanded from a screening test in trauma to being used by almost every medical specialty for diagnosis, monitoring or procedural guidance [1]. Much of this revolution was initiated in tertiary care centers; but with increasing pressure for expedited diagnosis and efficient use of manpower resources throughout healthcare, point-of-care ultrasonography (POCUS) has been adopted across the entire spectrum of clinical settings from outpatient clinics to critical care units. With its affordability, limited infrastructural, maintenance, and resource requirements, ultrasonography has an especially important role in environments where diagnostic imaging resources are limited. In such settings, ultrasound provides information that has a significant impact on patient outcomes and can change the way medicine is practiced [2, 3].

Pediatric emergency POCUS has been part of this movement, with published scanning protocols describing its use in the evaluation of trauma, abdominal pain [4, 5], dyspnea [6], and musculoskeletal complaints [7, 8], among others. This is much the same range of complaints that are the focus of adult emergency and critical care ultrasound. Indeed, it could be argued that the rationale for the use of clinician-performed ultrasonography is even more compelling in the care of children since the goal of minimal exposure to ionizing radiation is most important in this age group [9]. However, in contrast to adult emergency [10] and critical care medicine [11], there is currently no standard for the practice of pediatric emergency POCUS.

The following document is an initiation of this process. It is our hope that it will serve to define the field, establish standards of practice, delineate training requirements, and point to needed areas of scientific investigation. If adult emergency and critical care ultrasound are any guide, it is likely that an effect of this process will be the recognition of clinician-performed ultrasonography as a core competency of pediatric emergency care that requires a place in training programs at both post-graduate and undergraduate levels [12].

To develop this document, we assembled a group of thought leaders and content experts to review the scientific literature and use it to formulate evidence-based statements about emergency POCUS. For this initial effort, we assembled members of a pediatric emergency POCUS work group, which wrote the recently published POCUS guidelines for pediatric emergency physicians [13, 14]. With collaboration from additional content experts in the field, who had either completed an ultrasound fellowship or specialty training in pediatric emergency POCUS, we sought to create a comprehensive document summarizing the current evidence. For each application, the evidence is reviewed with respect to indications, current knowledge (or lack of it), curriculum objectives for learners, and identified pitfalls. We also developed standardized reporting guidelines for each ultrasound indication. The reporting guidelines are intended as templates that sonologists may choose to use as a method of extracting and documenting appropriate information about ultrasound exams for reporting, quality assurance, and if desired, research. They are not intended as stepwise instructions on how to complete exams. Each section was written by members of the core group of content experts and then edited by the lead authors (JRM, REL), and by Anthony J. Dean, MD, *Critical Ultrasound Journal* supervising editor for this piece.

Given the exponential growth of POCUS and descriptions of new exams, techniques, and indications, this document should not be viewed as a comprehensive summary of POCUS. Further, while we realize there is overlap between emergency medicine and other disciplines, such as critical care medicine, the purviews are different and the focus of this document is the pediatric emergency medicine provider. We recognize that a methodological limitation of this document is the absence of global representation among its authors, as well as a validated consensus process. Moreover, we acknowledge that many leaders in the field of POCUS, and particularly pediatric emergency POCUS are not included in our author group. Since this is the first step in an iterative process, we strongly hope that future versions of this document will draw more extensively on the expertise of pediatric emergency physicians worldwide. We hope that this document will provide a framework for both ongoing practice and serve as a springboard for continuing efforts in training and research.

## Diagnostic applications of ultrasound

### Ultrasound evaluation of the appendix

#### Evidence

1. Summary/brief explanation of indication
  POCUS has proven to be a valuable imaging examination in the evaluation of right lower quadrant abdominal pain to assess for findings of appendicitis.
2. Relevant adult-specific literature
  In a meta-analysis on the use of radiology ultrasound versus computed tomography (CT) for acute appendicitis, it was determined that CT had superior sensitivity (94 vs. 83 %) with similar specificity (93 vs. 94 %) [15].
  The American college of radiology (ACR) recommends CT over ultrasound in the routine evaluation of adults with suspected appendicitis [16].
  There have been several studies where emergency medicine physicians used POCUS to evaluate acute appendicitis. In 2000, Chen et al. examined 147 patients and reported a sensitivity of 96 % and specificity of 68 % with an accuracy of 89 %. This was significantly more favorable than the surgeon’s clinical accuracy of 71 % (*p* < 0.005) [17]. Fox et al., in 2008, using primarily emergency medicine residents for the POCUS evaluation, enrolled 132 patients and found a sensitivity of 65 % and a specificity of 90 % [18]. While the sensitivity was too low to recommend POCUS as a screening test for appendicitis, the specificity, similar to that reported in the radiology literature, suggested that a positive study could preclude further CT imaging. Similar results were reported by Mallin [19].
3. Relevant pediatric-specific literature
  In the radiology literature, sensitivity of ultrasound examination in pediatric patients ranges between 50–100 %, and specificity between 88–99 % [15, 20]. In a large multicentered study, ultrasound sensitivity matched that of CT when the patient had symptoms for greater than 48 h [21].
  In 2010, the ACR reaffirmed that ultrasonography should be the first-line imaging study in children under 14 years and in pregnant women [16].
  In a subanalysis of the study by Fox et al., the authors reported the sensitivity and specificity for the 2–17 year population (*n* = 42) to be 74 and 85 %, respectively [18].
  Out of concern for the radiation risks associated with (CT) [22, 23] several authors have proposed staged imaging strategies with ultrasound as the initial imaging study, followed by CT in equivocal or non-visualized studies [24–26]. This “sono-first” approach was found to be cost effective [27].
  Two recent studies have evaluated PEM physician conducting POCUS for appendicitis. Sivitz et al. [28], with 264 POCUS studies by PEM fellows and attendings, found a sensitivity of 85 % (95 % CI 75–95), specificity of 93 % (85–100), positive likelihood ratio of 11.7 (6.9–20), and negative likelihood ratio of 0.17 (0.1–0.28). Elikashvili et al. [29], looking at 150 patients also demonstrated the specificity of PEM-performed POCUS to be 94 % (95 % CI 88–97), indicating that a positive POCUS exam can be acceptable as a rule-in study. They also demonstrated a significantly decreased length of stay for patients with disposition by POCUS compared to radiology (154–288 min) without any cases of missed appendicitis.
  To address the limited availability of radiologic ultrasonography at one institution, one study evaluated use of teleultrasonography to diagnose appendicitis in children. Accuracy of emergency medicine resident-performed POCUS interpreted in real time by a remote expert was high and similar (sensitivity 100 %, specificity 98 %, positive predictive value 95 %, negative predictive value 100 %) to that of onsite expert-performed ultrasonography [30].
4. Outstanding questions to be answered/voids in the literature to date
  While the minimum appendiceal measurement for acute appendicitis is generally stated at 6 mm, in a study of healthy patients without any abdominal complaints, 23 % of subjects had an appendiceal diameter greater than 6 mm, and 9 % were greater than 7 mm [31]. Therefore, a measurement greater than 6 mm does not necessarily indicate acute appendicitis. Some have suggested using a diameter cutoff at 7 mm to avoid false-positive diagnosis [32].
  As can be seen from the previous discussion, published data on accuracy of POCUS for appendicitis are somewhat limited and inconsistent in their findings. There are no studies to our knowledge regarding the training that is needed to achieve competence in this application of POCUS.

#### Curriculum objectives

1. Describe the* indications* for POCUS evaluation of the appendix
  The indication for performing a right lower quadrant ultrasound is in a patient who presents with a history or examination concerning for acute appendicitis.
2. Describe the* limitations* of the POCUS evaluation of the appendix
  The primary limitation of sonography for appendicitis is the lack of consistent visualization of the normal appendix. Published visualization rates have ranged from 22 to 98 % [20].
  While increased body habitus has often been cited as a limitation for ultrasound, Sulowski et al. demonstrated that compared to normal weight children, there were no statistically significant differences in outcomes or CT utilization for obese children undergoing evaluation for acute appendicitis [33].
  Measurement of the appendiceal diameter alone may not be sufficient to diagnose appendicitis. The presence or absence of other variables such as appendiceal wall thickness, the presence of an appendicolith, free fluid, or periappendiceal inflammatory changes may also help to identify positive or negative cases [32, 34].
  It can be difficult to visualize a perforated appendix due to the inability to perform a graded compression exam on a patient with peritonitis. The presence and constellation of secondary findings such as complex fluid collections, dilated bowel and RLQ echogenic fat may help distinguish perforated appendicitis from uncomplicated appendicitis [35, 36].
3. Describe the* relevant anatomy* to be identified in the POCUS evaluation of the appendix
  Structures in the right lower quadrant that aid in identification of the appendix include the psoas muscle, iliac artery and vein, terminal ileum, cecum and ascending colon. The relationship between the psoas muscle and iliac vessels remains constant, as does the ascending colon being the most lateral right-sided intra-abdominal structure. However, the cecum’s position is not consistent in every patient, and may reside in the (normal) cecal fossa, or in a more cephalad, medial, or pelvic location. This can make finding the appendix difficult, as its origin off the medial portion of the cecum 1–2 cm from the ileoecal valve may be equally hard to find. In addition, finding the appendix with a normally positioned cecum may be compromised by a retro-cecal position.
  Performing the examination requires a graded compression technique, where gentle downward pressure is applied with the ultrasound transducer. This helps to visualize the non-compressible appendix by localizing the source of pain, as well as compressing away small bowel and displacing artifacts caused by bowel gas [37].
  The normal appendix is a blind ending, aperistaltic, tubular structure arising from the medial cecum, and measuring less than 6 mm in diameter. In the transverse orientation, it takes on a target-shaped appearance. A normal appendix appears round to ovoid and compressible [38]. When ovoid, the diameter should be measured along the narrow part of the oval in order not to overestimate the diameter. Measurements are taken from outer wall to outer wall.
  In acute appendicitis, the appendix will assume a rounded shape, and the diameter should be greater than 6 mm. Increasing wall thickness makes acute appendicitis increasingly likely and 1.7 mm (measured from hyperechoic mucosa to hyperechoic serosa) is used by some authors as a cutoff value [32].
  There may also be secondary signs of inflammation such as periappendiceal inflammation, free fluid, appendicolith, or hyperemia of the appendiceal wall [17].
  The sonographic appearance of the submucosal layer, ranging from sharply delineated to hazy to absent in advanced disease, has been suggested as a tool to grade acute appendicitis [39].
  In perforated appendicitis, a phlegmon or abscess may be seen, while the appendix may appear decompressed or may not be visualized [35, 36].
4. Recognize specific* pitfalls* involved with POCUS evaluation of the appendix
  Misidentifying normal small bowel or folds of the bowel wall for an appendix.
  Visualization of only the normal portion of a diseased appendix, where inflammation is isolated to the tip (false negative).
  Misdiagnosing a normal appendix as inflamed secondary to other intra-abdominal processes, such as Crohn’s disease or pelvic inflammatory disease (false positive).
  Misdiagnosing acute appendicitis based on a diameter greater than 6 mm in an ovoid appearing compressible appendix and/or without any secondary signs of inflammation.

### Ultrasound evaluation of the biliary tract

#### Evidence

1. Summary/brief explanation of *indications*
  The use of POCUS for hepatobiliary disease centers on identifying the presence of gallstones. A secondary goal is identification of evidence of biliary inflammation and/or biliary obstruction. Although still considered a rare disease in the pediatric population, rates have been on the rise over several decades [40]. This is thought to be due to increasing rates of obesity and better availability of diagnostic imaging [41, 42].
  Pediatric patients may present with atypical or intermittent symptoms, making misdiagnosis or delay in diagnosis common.
2. Relevant adult-specific literature
  Research in adult populations has shown that emergency medicine physicians can accurately perform and interpret gallbladder POCUS [43, 44].
  POCUS evaluation of the gall bladder can decrease emergency department length of stay, especially during times when department of radiology studies is not available (e.g., evening and nighttime hours) [45–47].
  Clinician sonologists who had performed over 25 biliary POCUS exams showed excellent agreement in image interpretation as compared with experienced sonologists [48].
  Patients are overwhelmingly satisfied with POCUS performed in the emergency department. A high percentage of patients would rather stay in the ED to have a POCUS performed than being transported to the radiology suite for the exam [49].
3. Relevant pediatric-specific literature
  In a recent case series of 13 pediatric patients with cholecystitis or biliary tract disease, no patient had the classic presentation of fever, elevated leukocyte count and an acute abdomen. POCUS may help avoid missing this important disease entity [50].
4. Outstanding questions to be answered/voids in the literature to date
  There are limited published data on the use of POCUS in the assessment of the biliary system of the pediatric patient.
  It is unclear how much training is required to develop competence in performing ultrasonography for acute cholecystitis in the pediatric population. In one study of adult patients, EM residents’ accuracy was similar to that of an experienced member of faculty [44]. In another study involving adult patients, performance of up to 50 emergency ultrasound examinations appeared to have little effect on the accuracy of right upper quadrant ultrasound [51]. In a third study as noted previously, sonologists who had performed over 25 ultrasounds showed excellent agreement in image interpretation as compared with experienced sonographers, with increasing accuracy correlating with increasing experience in those with less than 25 examinations [48]. Rather than simply requiring an arbitrary number of examinations, another method of competency assessment may be necessary [51].

#### Curriculum objectives

1. Describe the* indications* for biliary tract POCUS
  The primary indications for biliary tract POCUS are the identification of cholelithiasis and acute cholecystitis.
  Clinical indications for performing gallbladder ultrasound are symptoms, signs, or laboratory abnormalities that prompt concern for biliary tract disease. These include but are not limited to the following: patients presenting with upper abdominal pain or tenderness, nausea, vomiting fever, jaundice.
2. Describe the* limitations* of biliary tract POCUS
  Since it is a focused exam, POCUS is not intended to identify all abnormalities and pathologies of the right upper quadrant. POCUS should be interpreted in the context of the clinical picture and if the findings are non-diagnostic, further imaging studies may be warranted.
  Pathology of structures surrounding the gall bladder, such as the liver, pancreas or portal system, may not be identified by a focused exam.
  Biliary POCUS may be technically limited by overlying bowel gas, adipose tissue and patient discomfort.
3. Describe the* relevant anatomy* to be identified with biliary tract POCUS
  The gallbladder usually lies posterior to the inferior margin of the liver in the mid-clavicular line. In some patients, the fundus may extend several centimeters below the costal margin; in others, the gallbladder may be high in the porta hepatis surrounded by liver parenchyma.
  The gallbladder should be evaluated with the highest frequency range that provides adequate depth and penetration using an abdominal transducer. Images may be obtained subcostally or by looking through the rib spaces more superiorly.
  If these maneuvers do not provide adequate images, it may be helpful to place the patient in a left lateral decubitus position.
  When imaging the gallbladder with the transducer placed below the costal margin, a deep inspiration by the patient lowers the diaphragm and liver and may allow better visualization of the hepatobiliary structures.
  When imaging the gallbladder with the transducer placed between rib spaces, sonographic shadowing may be decreased by orienting the transducer parallel to the ribs directing the ultrasound beam through the intercostal spaces.
  A sonographic Murphy’s sign if present should be noted. It is obtained by eliciting tenderness reproducing the patient’s symptoms when probe pressure is applied directly over the gallbladder, with the absence of symptoms when probe pressure is applied either medially or laterally to the gallbladder.
  The gallbladder wall should be measured along the anterior wall between the lumen and the liver parenchyma. Measurement of the posterior wall may be inaccurate due to difficulty in delineating the outer wall of the gallbladder which usually abuts gas-filled intestinal structures. Posterior acoustic enhancement or the presence of gallstones may further degrade the precision of measurement of the posterior wall.
  Pericholecystic fluid may appear as an anechoic stripe seen along the anterior surface of the gallbladder or as a heterogeneously echogenic fluid collection adjacent to the fundus or posterior wall of the gallbladder. It may also appear as a hypoechoic region within the hepatic parenchyma adjacent to the wall of the gallbladder.
  Evaluation of the common bile duct may also be performed for abnormalities including dilatation and choledocholithiasis. The common bile duct usually lies anterolateral to the portal vein with the common hepatic artery lying anteromedial. Color Doppler may be used to help differentiate between the two. The common bile duct should be measured and evaluated for dilation.
  To determine whether gallstones are mobile or impacted in the gallbladder neck, the patient can be turned into the left lateral decubitus position or asked to sit up or stand from the supine position.
4. Recognize specific* pitfalls* involved with biliary tract POCUS
  Missing findings by not scanning through the entire organ in two orthogonal planes.
  Mistaking other fluid-filled right upper quadrant structures for the gallbladder. These structures may include the portal vein, the inferior vena cava, and hepatic or renal cysts. Scanning in two planes and being mindful of surrounding anatomy will reduce this possibility. In addition, looking for the “exclamation point” sign with the gallbladder as the exclamation and the right portal vein as the point will help to verify the anatomy.
  Mistaking loops of small bowel for a gallbladder containing gallstones. The wall of the bowel is anatomically very similar to the wall of the gallbladder, and bowel gas can cause intense shadowing. This misreading can be avoided by systematically scanning through the entire organ, demonstrating that it is cystic and not tubular, and searching for peristalsis.
  Mistaking the common hepatic artery for the common bile duct. The common bile duct usually lays anterolateral to the portal vein with the common hepatic artery lying anteromedial. The common bile duct has thin walls, and the hepatic artery has thicker and more echogenic walls. Color Doppler may help make the distinction, as there should be no detectable flow in the common bile duct. If the common bile duct is of normal diameter, the distinction is usually moot, since both vessels have similar diameter, which is significantly smaller than that of the portal vein.
  Failure to measure the gallbladder wall on the surface that abuts the liver.
  Failure to measure the gallbladder wall exactly at right angles to its surface. Such measurements will exaggerate the thickness of the gallbladder wall.
  Gallbladder wall thickening also occurs when the gallbladder is contracted. This is normal in the postprandial state.
  Many nonsurgical diseases also cause gallbladder wall thickening including malnutrition, renal failure, liver failure, congestive heart failure, hypoproteinemia, hypoalbuminemia, and in patients receiving total parenteral nutrition.
  The gallbladder may be difficult to identify in chronic cholecystitis, especially when filled with stones. A gallbladder filled with one large stone or with a collection of stones may create the “wall echo shadow” (WES) sign, a finding that may result in failure to identify the gallbladder or mistakenly identifying it as the duodenum or transverse colon.
  Small gallstones may be overlooked or confused with artifact from adjacent bowel gas. The entire gallbladder should be scanned in multiple planes and if not well visualized the patient repositioned to evaluate for mobility of gallstones. Common bile duct stones are often not be visualized and may be suspected only by the shadowing they cause.
  Cholesterol stones are smaller and less echogenic than calcium containing stones, and frequently float to the anterior wall of the gallbladder.
  Stones in the gallbladder neck may be overlooked due to edge shadowing artifact. Small gallstones, which are intrinsically the most difficult to identify, are the most likely to cause obstruction by becoming impacted in the gallbladder neck. This area should be imaged in several planes to avoid missing a stone.
  Pneumobilia and emphysematous cholecystitis may be identified by increased echogenicity due to air artifact in the gallbladder wall and biliary tree.
  For most of their natural history, most gallstones are asymptomatic. The presence of gallstones does not rule out other life-threatening emergencies of the thorax and abdomen.
  Bowel gas may prevent adequate examination. Slow, graded compression can be used, or the patient may be repositioned. If the study is still inadequate, the limitation should be documented and other methods of evaluation should be used.

### Ultrasound evaluation of bladder volume

#### Evidence

1. Summary/brief explanation of indications
  POCUS can be used to determine the presence of urine in the bladder before attempting urethral catheterization in infants and children.
  If the bladder appears empty by ultrasound, the clinician may defer the procedure until the child’s bladder contains urine, therefore, avoiding failed procedure attempts [52].
  POCUS assessment of bladder volume may confirm urine production or urinary retention.
2. Relevant adult-specific literature
  Ultrasound of the bladder can evaluate suspected urinary retention, measure post-void residual volume and evaluate for bladder stones [53].
  In adult patients, the following equation has been utilized as an estimation of bladder volume: depth (cm) × width (cm) × height (cm) × correction factor 0.75 = bladder volume (cc) [54].
3. Relevant pediatric-specific literature
  A successful urethral catheterization is considered a urine volume sufficient for urinalysis and culture, usually considered about 2–2.5 ml [55–57].
  Milling et al. defined the urinary bladder index in infants (<2 years) as the product of anterior–posterior and transverse bladder diameters expressed in centimeters squared. A value of 2.4 cm^2^ was found to correlate with a bladder volume of 2 ml. In this study, all infants with a bladder index greater than 2.4 cm^2^ had a successful catheterization attempt and all children who had a urinary bladder index less than 2.4 cm^2^ had a failed catheterization attempt [54].
  Two other studies demonstrated that the use of a POCUS estimation of bladder volume in infants led to increased success rates of emergency department urethral catheterization [56, 57]. These studies advocated repeating the ultrasound every 30 min until adequate urine volume was achieved before catheterization attempt.
  Bladder POCUS can also be used in older children to determine if they are making urine. Bladder ultrasound may provide additional information in patients who otherwise appear euvolemic, but have reports of a prolonged interval since their last void.
  Qualitative assessment of bladder volume by health care providers can be learned with a brief, 10 min training session [58]. Specialized bedside bladder scanners may also be used if a bedside ultrasound system is not available [52].
  Bladder volume in children >3 years of age can be estimated by (depth × height × width) × 0.68 + 4 [59].
4. Outstanding questions to be answered/voids in the literature to date
  Further study on this topic may include determining how performing POCUS for pediatric bladder volume assessment affects emergency department length of stay, time to antibiotic administration, and parental satisfaction.

#### Curriculum objectives

1. Describe the* indications* for POCUS for bladder volume assessment
  Indications for pediatric bladder volume assessment include:
  Evaluation for urine in the bladder prior to urethral catheterization attempts in infants or those children who are unable to provide a sterile clean-catch specimen.
  Assessment for urine production in children with a history of a prolonged time period since their last void.
2. Describe the* limitations* of POCUS for bladder volume assessment
  Determination of bladder volume becomes less accurate with small bladder volumes.
3. Describe the* relevant anatomy* to be identified when performing bladder volume assessment
  The bladder can be recognized as an anechoic, fluid-filled, structure residing in the anterior inferior pelvis. When empty, it is located immediately behind the pubic symphysis. With increasing distention, the dome of the bladder extends in a cephalad direction above the pubic symphysis.
  Measurements should be taken in three dimensions if possible, allowing for calculation of bladder volume. In infants, the urinary bladder index can be calculated based solely on a transverse and anteroposterior measurement in the transverse plane.
4. Recognize specific* pitfalls* with POCUS of the urinary tract
  Failure to place the transducer caudal enough on the lower abdomen to visualize the bladder and the pubic symphysis.
  Mistaking other fluid-filled pelvic structures (e.g., ovarian cyst) for the bladder.
  Failure to measure the maximal diameter of the bladder in any one view and, consequently, underestimating bladder volume.

*Discussion of ultrasound-guided suprapubic bladder aspiration can be found in the procedures section.

### Ultrasound evaluation of deep venous thrombosis

#### Evidence

1. Summary/brief explanation of indications
  Deep vein thrombosis (DVT) should be suspected in patients who present with limb pain and/or swelling and with risk factors such as trauma, prolonged immobilization, malignancy, congenital heart disease, hypercoagulable disorders (including neoplastic disease and nephrotic syndrome), pregnancy, or patients with indwelling catheters.
  DVT in can be detected using compression ultrasonography. The presence of clot is demonstrated by the inability to compress the vein with the ultrasound probe, while it is being sonographically visualized.
  POCUS is most commonly used to assess the deep venous systems, since these can give rise to pulmonary emboli. Deep venous systems include those of the lower extremity (femoral and popliteal veins), those of the upper extremity (subclavian, axillary, brachial veins), and the internal jugular veins.
  POCUS can also easily identify thrombosed superficial veins.
2. Relevant adult-specific literature
  A meta-analysis from 2008 estimated an overall sensitivity of POCUS of 95 % (95 % CI 87–99 %) and a specificity of 96 % (95 % CI 87–99 %) when radiology ultrasound was used as the reference standard [60].
  One study of 156 patients with a suspected lower extremity DVT demonstrated that POCUS had excellent agreement with that performed in the radiology department (kappa = 0.9), and patients were evaluated more than 2 h faster with POCUS [61].
  POCUS may yield high negative predictive value for DVT evaluation, avoid unnecessary anticoagulant treatment, and may be performed when radiology studies are not available with good accuracy [62, 63].
  In adults, proximal lower extremity DVT can be excluded by a limited compression study of just the common femoral and the popliteal veins. If these veins can be compressed and the patient has a negative d-dimer study, DVT is excluded. If the veins can be compressed and the d-dimer study is positive, the patient requires another ultrasound test in 3–5 days to assess for distal venous thrombosis [64].
3. Relevant pediatric-specific literature
  To date, there is only a case series of POCUS utilized in the pediatric ED to evaluate for DVTs [65].
4. Outstanding questions to be answered/voids in literature to date
  There is limited description of POCUS for the diagnosis of DVT in children. All comparative studies between POCUS and radiology studies have been performed in adult patients. Assessing accuracy of POCUS for pediatric DVT will be difficult given that it is a rare entity in emergency departments. However, the technique has been shown to be simple and rapid to perform in adults and can be easily applied to children.

#### Curriculum objectives

1. Describe the* indications* for the POCUS evaluation for DVT
  Indications: DVT POCUS should be considered in patients with risk factors for DVT, who present with extremity pain, swelling, and/or shortness of breath.
  Risk factors for DVT include indwelling central venous catheters, congenital heart disease, inherited or acquired hypercoagulable disorders, history of pregnancy or malignancy, or history of trauma or medical illness requiring immobilization or hospitalization.
2. Describe the* limitations* of the POCUS evaluation for DVT
  Upper extremity and thoracic DVTs may be difficult to visualize on ultrasound in patients with indwelling catheters due to rib shadowing and air in the thorax.
  Clavicles and ribs prevent compression assessment of the proximal internal jugular, subclavian, and thoracic veins.
  Abdominal contents prevent compression assessment of the Iliac veins and the IVC.
  Imaging and compression of veins in patients with a high body mass index may be more challenging.
3. Describe the* relevant anatomy* to be identified with POCUS evaluation for DVT
  Compression ultrasonography verifies venous patency using an ultrasound transducer to compress and obliterate the vein lumen under direct visualization [66]. Comparison of contralateral sides may assist in diagnosis, as can the use of color and spectral Doppler.
  Pressure is applied through the transducer until the adjacent artery is seen to start to compress. If the vein does not compress with this amount of force, it is deemed noncompressible.
  Femoral vein: Evaluation starts at the inguinal ligament with compression every centimeter until 1–2 cm beyond its bifurcation into superficial and deep branches [66].
  Popliteal vein: Evaluation starts with vein compression superior to the popliteal crease and continues until the trifurcating terminal branches of the popliteal veins are identified [66]. The bony landmarks of the femoral condyles and the tibial plateau should be identified to confirm that the popliteal vessels are indeed being examined. When examined from the popliteal fossa, the popliteal artery lies immediately superficial/posterior to these bony landmarks, and the popliteal vein is immediately superficial/posterior to the artery.
  Internal jugular vein: compression starts at the level of the clavicle and continues superiorly until the base of the skull [66].
  The veins of the upper extremity to be assessed by compression include the axillary, brachial, and the antecubital.
4. Recognize specific* pitfalls* involved with POCUS evaluation for DVT
  Misidentifying lymph nodes or arteries as non-compressible veins.
  Mistaking a noncompressible vein for an artery.
  Mistaking large compressible collateral superficial veins for deep veins, when the deep veins are actually occluded by thrombus.
  Assuming that the presence of color flow or spectral Doppler signal rules out DVT. Many DVTs do not completely occlude the vein, hence the need for compression evaluation.
  Assuming that acute thrombus will be echogenic and, therefore, directly visualized on ultrasound. Acute thrombus is anechoic, hence the need for compression evaluation. With the passage of time, some thrombus becomes calcified and thus identifiable on B-mode with appropriate gain adjustments.
  Possible clot dislodgement during evaluation.
  Evaluation of chronic DVTs is an advanced skill that should be done by a vascular lab or radiology department with access to previous imaging or with scanning protocols that evaluate chronic DVTs.

### Ultrasound evaluation of the eye

#### Evidence

1. Summary/brief explanation of indication
  POCUS evaluation of the eye (ocular POCUS) allows visualization of the components of the globe and the optic nerve. Many pathologies can be diagnosed using ultrasound including foreign body, retinal detachment, vitreous hemorrhage, and lens dislocation [67]. Ocular POCUS can also be used in the evaluation of patients with signs and symptoms of increased intracranial pressure (ICP).
2. Relevant adult-specific literature
*Intra-ocular pathology* Ocular ultrasound is widely utilized in the ophthalmologist’s diagnostic evaluation of eye pathology. Several case reports describe an array of conditions diagnosed with emergency physician-performed POCUS. Blaivas et al. found that POCUS by emergency physicians had a sensitivity of 100 % and specificity of 97.2 % in the detection of ocular pathology when compared with computed tomography (CT) and evaluation by the ophthalmology service [68]. A meta-analysis found ocular POCUS to have excellent sensitivity of 97–100 % and specificity of 83–100 % in the diagnosis of retinal detachment [69].
*Optic nerve sheath diameter measurement* In addition to assessment of intra-ocular pathology, changes in the optic nerve sheath diameter (ONSD) correlate with increased ICP. Small adult studies have shown excellent sensitivity and specificity of ONSD measurements in the detection of increased ICP in both the emergency department and intensive care unit [70–73].
3. Relevant pediatric-specific literature
*Intra-ocular pathology* There are limited data regarding the use of emergency physician-performed POCUS to evaluate for intra-ocular pathology in children. An isolated case report describes the diagnosis of retinal detachment in a pediatric patient with POCUS in the emergency department [74]. Additionally, POCUS identification of optic drusen, retinoblastoma, and retinal hemorrhage in the setting of abusive head trauma has been reported [75–77].
*Optic nerve sheath diameter measurement* Pediatric normal values for the measurement of optic nerve sheath size have been established and several publications have described the use of these measurements in the evaluation of children at risk of increased intracranial pressure (ICP) in neurosurgical clinics [78–81]. A case–control study noted a significant difference between the ONSD of patients with signs of increased ICP diagnosed by CT when compared to controls [82]. Studies of ED physician-performed ONSD measurements are limited to small prospective case series regarding the use of POCUS in pediatric patients undergoing evaluation for increased ICP [83, 84]. Using CT or invasive measurement techniques as the reference standard, one study found a sensitivity of 83 % and specificity of 38 % of ONSD measurements performed by emergency medicine physicians and interpreted by ophthalmologic ultrasonographers. In this study, when ONSD measurements were performed and interpreted by emergency physicians, they found a sensitivity and specificity of 96 and 38 %, respectively. Further study focused on ONSD measurements in patients with suspected pediatric ventriculoperitoneal shunt failure and found ONSD measurements to have sensitivity of 61.1 % and specificity of 22 % for detecting shunt malfunction [85]. A study of children with hydrocephalus found utility in using a patient’s own baseline ONSD measurements as a comparison for when they are symptomatic, particularly when there are no changes noted with neuroimaging [86].
4. Outstanding questions to be answered/voids in the literature to date:
  Further studies are needed to evaluate the role of ocular POCUS in the assessment of ocular pathology and increased ICP in the pediatric population as current literature has demonstrated variable results.

#### Curriculum objectives

1. Describe the* indications* for ocular POCUS
  Indications for ocular POCUS include, but are not limited to: change in visual acuity, ocular pain, foreign body, eye trauma, and concern for increased intracranial pressure.
2. Describe the* limitations* of ocular POCUS
  Post-surgical changes or congenital anomalies of the eye may create scarring that prevents the expected changes in size in the optic nerve sheath with increase in intracranial pressure.
  To date, there are limited data regarding the accuracy of POCUS for measuring ONSD as a proxy for intracranial pressure. One study suggests a high sensitivity but suboptimal specificity.
  Differentiation of vitreous detachment from retinal detachment has been shown to be difficult for novice ultrasonographers leading to inaccurate diagnosis of retinal detachment [87].
3. Describe the* relevant anatomy* to be identified with ocular POCUS
  The anterior and posterior segments of the eye and the optic nerve should be examined. The cornea, the most anterior structure of the anterior segment, is identified as a thin, hypoechoic tissue superficial to the anechoic fluid of the anterior segment. The lens, located posteriorly within the anterior segment, is an anechoic thin structure flanked by the iris and the ciliary body, which are seen as echogenic structures extending centrally from the lateral walls of the globe. Deep to the lens, the posterior segment is filled with anechoic vitreous. The posterior wall of the globe contains the retina and choroidal layers that are not well visualized in the normal eye. Behind the globe, the optic nerve is a hypoechoic, elongated structure extending posteriorly and, in the evaluation of ONSD, is measured 3 mm from the interior surface of the optic cup.
4. Recognize specific* pitfalls* with ocular POCUS
  The measurement of the optic nerve sheath requires a scanning plane in the middle of the nerve (to avoid underestimation of diameter). Images that are low in quality or resolution can lead to inaccurate measurements.
  Ocular POCUS after traumatic injury to the eye or face should be performed without applying any transducer pressure to the eyeball. If there is a possibility of globe rupture, a thick layer of gel should be used to prevent contact between the eyelid and transducer. A study of 40 patients demonstrated small, transient elevation in intraocular pressure during ocular POCUS, but pressure change was similar to that caused by physical exam [88].
  POCUS may be limited in its ability to diagnose retinal detachment; therefore, sonologists should be cautious in ruling out the diagnosis.

### Ultrasound evaluation of abdominal trauma-focused assessment with sonography for trauma (FAST)

#### Evidence

1. Summary/brief explanation of indications
  The focused assessment with sonography for trauma (FAST) examination seeks to detect intraperitoneal fluid (hemoperitoneum), pericardial fluid (hemopericardium), and intrathoracic fluid (hemothorax) in patients after blunt or penetrating truncal trauma. The “extended” FAST (e-FAST) examination also assesses the chest for pneumothorax.
2. Relevant adult-specific literature
  In adults when CT scan is readily available a large meta-analysis determined that there was no demonstrable benefit in mortality with the use of ultrasound in blunt abdominal trauma [89].
  In two randomized controlled trials, the FAST examination safely decreased abdominal CT use in injured adults [90, 91] as well as decreased the time to the operating room, hospital length of stay, and complications [90].
  Evidence indicates that both emergency medicine physicians and surgeons can accurately perform the FAST examination [92–94].
  Competency in performing and interpreting the FAST examination may be accomplished after performing 10–30 examinations in adult patients [95–97] with error rates as low as 5 % [96].
  Lung ultrasound is more accurate than supine anterior chest radiograph for diagnosing pneumothorax [98].
3. Relevant pediatric-specific literature
  The sensitivity of the FAST examination for hemoperitoneum in pediatric trauma patients is variable, ranging from 31–100 % [99–101]. The sensitivity improves in the most severely injured patients [102]. This suggests that an ED evaluation protocol using abdominal ultrasound in children may have clinical utility. Patient sampling, selection bias, ultrasound protocols, and outcome definitions were highly variable in prior pediatric studies and account for the reported variation in test characteristics [101].
  The results of a meta-analysis of 25 observational studies of 3838 children demonstrate that FAST has a sensitivity of 80 % when used to identify those with hemoperitoneum [101]. The sensitivity of FAST, however, drops to 66 % when those patients with intra-abdominal injuries, but without hemoperitoneum, are included in the outcome of interest. Failure to identify patients with intra-abdominal injury without hemoperitoneum is a known limitation of the FAST examination. Furthermore, imaging the solid organs in addition to evaluating for intraperitoneal fluid resulted in only a modest increase in ultrasound sensitivity.
  The sensitivity of the FAST examination for children undergoing operative intervention or blood transfusion for their abdominal injury is 88 % [103].
  FAST examination may be incorporated into other aspects of the trauma evaluation to improve the accuracy of the test. One small study found that when combined with an AST or ALT > 100 IU/L the specificity of the FAST examination was 98 %, suggesting a negative FAST and transaminases <100 IU/L should result in patient observation instead of abdominal CT scanning [104].
  Observational data demonstrate that the FAST examination has limited impact on abdominal CT use in injured children at very low (<1 %) and very high risk (>10 %) of intra-abdominal injury. However, use of the FAST examination in children considered to have 1–10 % risk of intra-abdominal injury safely decreased abdominal CT use [105]. In addition, surgeons desire for abdominal CT scanning decreases once a FAST examination has been obtained [103].
  The only evidence in children for pneumothorax detection with ultrasound is a case series describing needle placement for treatment of the pneumothorax [106].
4. Outstanding questions to be answered/voids in the literature to date
  Limited published data exist on pediatric FAST examinations as a screening tool. Currently, no published randomized control trials of the FAST examination in pediatric patients exist.
  The role of contrast-enhanced FAST to improve detection of solid organ injury in children requires exploration.
  There are limited data on test characteristics of POCUS for diagnosing pneumothorax in pediatrics.

#### Curriculum objectives

1. Describe the* indications* for the FAST examination.
  Indications for the FAST examination include:
  For hypotensive pediatric trauma patients, the FAST examination can rapidly identify hemoperitoneum, hemothorax, or cardiac tamponade and expedite operative intervention and/or transfusion of blood products.
  In hemodynamically stable patients, a positive FAST examination will typically require further evaluation with abdominal CT scan. If one cannot be obtained in a timely manner or the patient becomes unstable, serial FAST examinations can be performed to determine active bleeding by assessing for increasing amounts of intraperitoneal fluid.
  In hemodynamically stable patients with negative FAST examinations, abdominal CT scans may be obtained depending on risk factors for intra-abdominal injury [107], or patients may be followed by serial FAST examinations.
2. Describe the* limitations* of the FAST examination
  The FAST examination does not effectively evaluate solid organs for injuries. In addition, approximately, 25 % of intra-abdominal injuries in children do not have hemoperitoneum [107], thus the FAST examination may be normal despite the presence of an intra-abdominal injury.
  False-positive FAST examinations (up to 4 % of FAST examinations [101]) may lead to abdominal CT scans that would otherwise not have been obtained.
3. Describe the* relevant anatomy* to be identified with the FAST examination
  The FAST examination utilizes four specific locations to evaluate for intraperitoneal, intrathoracic, and pericardial fluid. The protocol includes views of the right and left upper quadrant (RUQ, LUQ), pelvis and heart.
    1. In the RUQ, a thorough examination visualizes from the diaphragm superiorly to the inferior portion of the liver. Visualization above the diaphragm evaluates for right-sided hemothorax. Morison’s pouch (interface of the liver and kidney) is the dependent space of the upper abdomen in the supine position and the most common location to identify hemoperitoneum in adults [108]. Imaging the inferior pole of the right kidney evaluates for fluid in the paracolic gutter.
    2. The LUQ is evaluated similar to the RUQ. A thorough examination should visualize from the diaphragm to the inferior portion of the spleen. Visualization above the diaphragm evaluates for left-sided hemothorax. The splenorenal space differs from Morison’s pouch due to the configuration of the splenorenal ligament as well as the relatively small size of the spleen compared to the liver. Depending on whether hemorrhage in the left upper quadrant is occurring inside or outside of the lesser sac, free fluid may accumulate circumferentially around the spleen, below the diaphragm (subphrenic), at the inferior pole of the spleen, or in the splenorenal fossa. Visualization of the inferior pole of the left kidney evaluates for hemorrhage in the left paracolic gutter.
    3. The pelvis is visualized for intraperitoneal fluid with both transverse and sagittal views. The urinary bladder is used as a landmark to evaluate for hemoperitoneum. Intraperitoneal fluid may accumulate superiorly and/or posteriorly to the bladder. The pelvis is the most common location for free fluid in pediatric patients [109].
    4. The FAST examination of the heart is primarily directed to the detection of intra-pericardial fluid. The most common FAST views make use of the subxiphoid window. The image displayed includes the liver and the heart. If the subxiphoid view is inadequate or difficult to obtain, the parasternal long axis view may be used.
  The e-FAST examination includes imaging the chest to evaluate for pneumothoraces
    The transducer (a linear is usually used) is placed anteriorly in the mid-clavicular line in a longitudinal plane. The lungs should be visualized from the diaphragm to the clavicles.
    If lung sliding or B lines or lung pulse are seen, pneumothorax is excluded; underneath the transducer at this point of the chest. If all 3 of these are absent, pneumothorax may be present. In addition, the presence of a lung point (also referred to as the “leading-edge”) is pathognomonic for the presence of pneumothorax.
    Color Doppler and motion mode (M-mode) can be used as adjuncts for evaluating lung sliding.
4. Recognize specific* pitfalls* involved with the FAST examination.
  Pitfalls to avoid when performing the FAST examination include the following:
  Mistaking the IVC, aorta, gallbladder or intraluminal intestinal fluid as intraperitoneal fluid (false positive).
  Interpretation of ascites, pleural effusion, or pericardial effusion as being due to traumatic hemorrhage. In most cases, there are clinical or historical clues to the correct diagnosis.
  Failure to identify all spaces within a given region as described above and concluding that the examination is negative (false negative).
  Failure to reduce the gain when evaluating the pelvis, and, therefore, being unable to identify intraperitoneal fluid behind the bladder due to posterior acoustic enhancement (false negative).
  Failure to utilize parasternal views, when unable to visualize the heart via the subxiphoid window.
  Pitfalls to avoid when performing the e-FAST examination include the following:
  Incomplete evaluation of the lungs concluding the exam is negative for pneumothorax (false negative).
  Interpreting lack of lung sliding due to pleural adhesions or right mainstem intubation as pneumothorax (false positive). Frequently, in such situations the presence of lung pulse and/or B-lines points to the absence of pneumothorax.
  Failing to identify free fluid in the pelvis due to the posterior acoustic enhancement overlying the area behind the bladder

### Ultrasound evaluation of the heart and inferior vena cava

#### Evidence

1. Summary/brief explanation of indications
  Focused cardiac ultrasound (FOCUS) [110, 111], which may include the evaluation of the heart as well as the inferior vena cava (IVC), is a limited focused clinician-performed cardiac evaluation directed to identifying specific cardiac disease states in addition to assessing the functional condition of the heart. FOCUS is used in a wide range of clinical conditions and not limited to diagnosing cardiac pathology. Specifically, FOCUS is used to evaluate the functional assessment of the heart in the setting of various shock states to guide appropriate management. FOCUS may fail to identify pathological findings revealed by comprehensive pediatric echocardiography [112] or targeted neonatal echocardiography [113]. FOCUS is not intended to supplant these extended comprehensive examinations.
  FOCUS can be utilized to rapidly assess global cardiac systolic function in critically ill patients with tachycardia, hypotension or dysrhythmias. Evaluating global cardiac systolic function may help to differentiate cardiac from other causes of hypoxia or shock [110, 111, 114, 115].
  Assessing pericardial effusion by FOCUS is critical for the evaluation of suspected cardiac tamponade in both the nontraumatic and trauma setting [115–120].
  The international pediatric basic and advanced life support recommendations state that “FOCUS may be considered to identify potentially treatable causes of a cardiac arrest, but the benefits must be carefully weighed against the known deleterious consequences of interrupting chest compressions” [121] and was reaffirmed by an international consensus panel of experts [110].
  Evaluation of the inferior vena cava can estimate intravascular volume status [110, 111].
  Serial FOCUS exams can help to gauge the patient’s response to resuscitative interventions, such as fluid boluses, inotropic support and pericardiocentesis [110, 111].
2. Relevant adult-specific literature
  One of the first uses of FOCUS was to evaluate the heart for pericardial effusion as part of the Focused Assessment with Sonography for Trauma (FAST) examination [115].
  Several case series have suggested that emergency department echocardiography in penetrating trauma patients is sensitive for identification of cardiac injuries and leads to rapid diagnosis and improved survival [115, 116].
  Emergency physicians have also demonstrated the ability to accurately detect pericardial effusions not secondary to penetrating trauma [117–119].
  With limited training, emergency physicians have been shown to accurately characterize left ventricular systolic function in hypotensive patients [122].
  Several studies have shown that emergency physicians can determine hemodynamic parameters, like ejection fraction and other hemodynamic information comparable to data obtained with comprehensive echocardiography [122, 123].
  FOCUS may be helpful in cardiac arrest scenarios to help predict outcomes of resuscitative efforts, with a lack of sonographic cardiac activity indicating poor survival [124, 125].
  Unlike more comprehensive sonographic cardiac assessments, FOCUS can be rapidly deployed and incorporated into advanced life support algorithms without prolonging interruptions in chest compressions, delaying medications or other cardiopulmonary resuscitation measures [125–128].
  In the adult with hypotension, ultrasound measurements of the inferior vena cava (IVC) have been used to estimate central venous pressure [127]. Dynamic assessments of the IVC have shown that with inspiration complete collapse of the IVC may indicate hypovolemia, while decreases in diameter of less than 50 % may represent fluid overload or increased right atrial pressures [129–131]. Other data from studies in trauma patients suggest that the aorta to IVC ratios may be a more reliable measure of hypovolemia [132]. Dynamic evaluation of the IVC in conjunction with thoracic ultrasound can help with assessing fluid responsiveness for patients in shock [133].
  There are various arithmetic formulas used for the assessment of IVC collapse, the most common being the collapsibility index [(IVC max diameter- IVC min diameter)/IVC max diameter]. Some have advocated for using clinical gestalt of IVC collapse to estimate volume status [134] (Fields JM, AEM).
3. Relevant pediatric-specific literature
  In a survey from 2008, 61 % of pediatric emergency departments reported using ultrasound clinically, with 59 % specifying evaluation for cardiac activity, 59 % for pericardial effusion and 7 % using POCUS for advanced cardiac applications [135].
  Literature describing the use of POCUS in detecting significant cardiac pathology in children such as cardiac tamponade, dilated cardiomyopathy from myocarditis, congenital heart disease and infective endocarditis has been described in a number of case reports [128, 136–141].
  Pediatric critical care and pediatric emergency medicine physicians with focused training were able to diagnose pericardial effusions, cardiac contractility abnormalities, and left ventricular enlargement with an accuracy of 91 % [142, 143].
  Studies have shown that pediatric emergency medicine physicians with POCUS training are both reliable and accurate in assessing left ventricular function and preload by estimating IVC collapsibility when compared to cardiologists/echocardiographers [144, 145].
  The literature on FOCUS evaluation during cardiac arrest in pediatrics is limited to case reports or series. One series of fourteen patients showed good correlation between the presence or absence of a pulse on physical examination and the presence or absence of cardiac activity on ultrasound. [128].
  Both the ratio of aorta to IVC and the dynamic assessment of IVC collapsibility with inspiration have been investigated in the pediatric population as assessments of hydration status and both metrics may correlate with fluid status [146–148].
4. Outstanding questions to be answered/voids in the literature to date
  More research is needed to create an evidence-based sonographic assessment for pediatric shock and undifferentiated tachycardia similar to guidelines developed for adult patients [149].
  More evidence is needed on the use of POCUS during pediatric resuscitation [110, 128].
  Normal values for different ages, disease states and populations are still needed for sonographic identification of fluid status in children, as well as investigations into the utility and impact of incorporating ultrasound into clinical pathways for dehydration [146–148].
  Investigation is needed into the potential use of communications technology to facilitate real-time tele-echocardiography/POCUS image transfer for consultation with pediatric intensivists or resuscitation experts in critical care or emergency scenarios [110, 150–152].

#### Curriculum objectives

1. Describe the* indications* for FOCUS
  FOCUS should be considered for patients with signs of potential cardiac pathology such as shortness of breath, chest pain, syncope, hypotension/shock, a new murmur, and cardiac arrest.
  The use of FOCUS in infants and children by physicians is still in its investigatory phase. As more outcome-driven evidence is attained, indications are likely to evolve. At the current time, FOCUS may assess for the following:.
  Assessment of cardiac contractility, pericardial effusion, and hypovolemia, in the setting of undifferentiated shock or tachycardia.
  Diagnosis of pericardial effusion and tamponade as part of the FAST exam, and in non-traumatized patients.
  Intravascular volume assessment of patients with hypovolemia secondary to vomiting and diarrhea.
  Assessment of cardiac motion and/or reversible causes of PEA in cardiac arrest.
2. Describe the* limitations* of FOCUS
  FOCUS is not primarily directed to the diagnosis or exclusion of congenital heart disease or its complications.
  Cardiovascular assessment/windows may be limited by injuries, dressings, or body habitus (including cachexia, obesity, scoliosis, and contractures).
  Standard measurements of the IVC/aorta are not well established for all age groups. Serial exams may be more useful to guide resuscitation than an exam at a single point in time.
  Inotropic medications and positive pressure ventilation may affect the size and elasticity of the IVC.
  Foreshortening may distort the normal circular appearance of the left ventricle in short-axis views leading to incorrect estimation of LV function.
3. Describe the* relevant anatomy* to be identified with FOCUS
  Relevant anatomy is dependent on the indication for FOCUS and the question to be answered.
  Standard cardiac assessment includes views in the sub-xiphoid, parasternal long-axis, parasternal short axis and apical four-chamber windows with additional views as necessary, such as suprasternal views and extended apical views [110].
  The IVC is usually assessed in a sub-xiphoid longitudinal plane as it travels through the liver parenchyma, crosses the diaphragm and enters the right atrium. Dynamic assessment may include measurements of vessel diameter during inspiration and expiration using m-mode. The descending aorta is typically viewed in its sub-xiphoid location and measurements of maximum size during systole are compared to the IVC in the transverse plane, usually at the level of the renal arteries.
4. Recognize specific* pitfalls* involved with FOCUS.
  Pitfalls to avoid when performing cardiac FOCUS include the following:
  Prolonged pauses in chest compressions for more than 10 s while performing FOCUS during pediatric cardiac arrest [110, 114, 128].
  Misinterpreting pericardial fat as a pericardial effusion or pleural effusions as pericardial effusions.
  Positive pressure ventilation and vasopressors may have variable impact on the size and respiratory variation of the IVC [153, 154].
  Mistaking the IVC for the Aorta during the assessment of volume status. The aorta can be identified based on the knowledge of anatomical branching, different flow pattern on pulsed wave Doppler, and a thicker pulsatile wall.
  In the presence of a pericardial effusion, severe tachycardia can impede the identification of diastolic collapse of the right ventricle and/or collapse of the right atrium, resulting in failure to diagnose tamponade. Although not routinely a part of the FOCUS exam, other methods for assessment of cardiac tamponade may be used, including M-mode assessment of respiratory variations in left ventricular diameter, Doppler assessment of mitral inflow velocity variation and visualization of a plethoric IVC [155].
  Confusion of right and left heart chambers and major aortic arch vascular branches may occur due to improper transducer orientation, especially in the setting of congenital heart disease [110, 156].

*Discussion of ultrasound-guided pericardiocentesis can be found in the procedures section. 

### Ultrasound evaluation of intussusception

#### Evidence

1. Summary/brief explanation of indication
  Ultrasound has become the diagnostic test of choice in the evaluation of patients with suspected *ileocolic* intussusception.
  In addition to making the diagnosis of intussusception, ultrasound can be utilized to determine if blood flow is still present to the affected bowel, or to identify free fluid, which may prognosticate the success of enema reduction.
2. Relevant adult-specific literature
  While there are stark differences between the pathophysiology, presentation, and treatment of intussusception in adults and children, the sonographic findings remain the same. In one illustrative case report, an EM physician recognized classic signs of intussusception in a patient with chronic abdominal pain [157].
3. Relevant pediatric-specific literature
  The use of ultrasound in the diagnosis of intussusception was initially described in 1977 [158]. Since then, the safety, ease, and accuracy of sonography have largely replaced plain radiographs as the initial screening modality [159, 160]. Sonography has since reported sensitivities of 98–100 % and specificities of 88–100 % when performed by imaging specialists [161–164].
  Radiologists have noted that even junior residents can interpret the typical sonographic findings, often as well as their supervising attendings [161, 164].
  There has been a single prospective, observational study involving POCUS for the evaluation of intussusception. Eighty-two patients underwent POCUS as well as ultrasound in the department of radiology. POCUS demonstrated a sensitivity of 85 % (95 % CI 54, 97 %) and a specificity of 97 % (95: 89, 99 %), suggesting that POCUS could be used to rule in a diagnosis of intussusception [165].
4. Outstanding questions to be answered/voids in the literature
  Prospective studies from pediatric emergency centers are needed to determine what level of training is adequate to become competent in the use of bedside sonography as a screening tool for intussusception.
  Evaluation is needed into outcome measures such as time to fluoroscopic reduction and emergency department length of stay when POCUS is used for the evaluation of intussusception.
  With only a single observational study to date, further investigation is needed to characterize the performance characteristics of POCUS assessment of intussusception in different emergency settings.

#### Curriculum objectives

1. Describe the* indications* for POCUS for the evaluation of intussusception
  POCUS is indicated for the evaluation of children presenting with clinical findings concerning for the presence of an *ileocolic* intussusception. These may include all or some combination of the following: vomiting, bloody or guaiac-positive stool, “currant jelly stool,” colicky abdominal pain, or a sausage-shaped palpable mass on the *right side* of the abdomen.
2. Describe the* limitations* of POCUS for intussusception
*Ileocolic* intussusceptions may spontaneously reduce before or after the POCUS examination. In the former case, this will result in a missed diagnosis. In the latter case, a positive ultrasound examination will be followed by a negative barium or air enema study [166].
  Positive identification of an intussusception may not address the presence of a pathologic lead point. These should be considered in patients who present outside the typical age range, or with a suggestive history or physical examination findings.
3. Describe the* relevant anatomy* to be identified in the POCUS examination for intussusception
  The primary area of concern for an ileocolic intussusception is the ascending colon found in the right lateral abdomen. This is imaged in transverse and sagittal planes from the hepatic flexure down to the area of the ileocecal valve in the right lower quadrant. A normal appearing ileocecal valve rules out an ileocecal intussusception. When normal appearing colonic anatomy is not found, a further search along the path of the transverse and descending colon may be undertaken.
  Sonographically, the intussusception has been described as a “target” or “doughnut” in transverse view, and as a “pseudokidney,” or “hayfork” in an oblique or longitudinal view.
  Small bowel intussusceptions may be encountered and should be distinguished from ileocolic intussusceptions. Beyond recognizing a normal appearing cecum, small bowel intussusceptions are also smaller and shorter in length.
  Further evaluation of the intussusception is directed at the identification of signs suggesting advanced disease and, consequently, the unlikelihood of successful enema reduction. These include loculated free fluid surrounding the intussusception, or “interloop” fluid within the intussusception. These findings are suggestive of bowel wall injury [167, 168]. Free intraperitoneal fluid has been noted with intussusception and is not associated with poor outcomes [169].
  Another sign of bowel wall ischemia is the absence of blood flow on color Doppler imaging. Several authors compared groups of patients with intact and absent flow, and found a decrease in the success of enema reduction in those patients without color flow [170, 171]. This is, however, not necessarily an absolute contraindication for attempted enema reduction [172].
  The presence of echogenic foci has been described in large bowel intussusceptions as well as in necrotizing enterocolitis [173, 174]. These foci, which are representative of air within the bowel wall, are suggestive of reduction failure and perforation.
4. Recognize specific* pitfalls* involved in the POCUS examination for intussusception.
  Pitfalls to avoid when performing a sonographic examination for intussusception include the following:
  Mistaking thickened bowel loops, stool or other abdominal masses for an intussusception.
  Imaging the bowel in only one plane or incompletely visualizing the posterior wall increases the chances of misinterpreting other abdominal masses as an intussusception. Always look for the “target sign” in cross section and the “pseudokidney” in long axis.
  Not recognizing the presence of poor prognostic findings prior to attempted enema reduction.
  Color Doppler assessment should only be performed by sonologists with experience in its use

### Ultrasound evaluation of the lung

#### Evidence

1. Summary/brief explanation of indications
  POCUS can be incorporated into the evaluation of pediatric patients presenting with respiratory distress and/or hypoxemia to assess for the presence of pneumothorax, hemothorax, or pleural effusion [175–177].
  The lungs can be evaluated sonographically in pediatric patients presenting with any respiratory symptoms or complaints to differentiate between pneumonia and bronchiolitis/viral pneumonia, as well as parapneumonic pleural effusion vs. empyema [175, 177].
2. Relevant adult-specific literature
  POCUS detection of a pneumothorax in emergency department and intensive care unit patients has become highly prevalent, due to its high sensitivity and specificity [175, 178–182].
  Pleural effusions: The sensitivity of POCUS for identifying pleural fluid has been shown to be 92 % with a specificity of 93–97 % [182]. POCUS can be used for thoracentesis-guidance/assistance of pleural effusions accurately [182].
  Pneumonia: Several studies have looked at the ability of POCUS to detect pneumonia in the setting of critically ill patients. Results show a sensitivity of 88–90 % and a specificity of 95–98.5 % using CT as the reference standard [183, 184]. A study done by ED physicians demonstrated that POCUS had a sensitivity of 96.9 % (31/32) for diagnosing pneumonia, compared with that of only 75 % (24/32) for chest radiograph (CXR), with CT confirming pneumonia in 8 US-positive CXR-negative patients [185].
3. Relevant pediatric-specific literature
  Pneumonia: There have been studies and a meta-analysis that examined the diagnosis of pneumonia by POCUS with high sensitivity and specificity [185–193]. An Italian study demonstrated that of 79 children with suspected pneumonia, 60 had pneumonia detected by ultrasound and 53 had CXR diagnosis of pneumonia; 4/7 which were US-positive and CXR-negative had CT confirmation of pneumonia and 3/7 had a clinical course consistent with bacterial pneumonia [186]. Another study of POCUS demonstrated 86 % sensitivity and 97 % specificity for detection of pneumonia confirmed by CXR [189]. A meta-analysis demonstrated a pooled sensitivity of 96 % (95 % CI 94–97 %) and specificity of 93 % (95 % CI 90–96 %); positive likelihood ratio of 15.3 (95 % CI 6.6–35.3) and negative likelihood ratio of 0.06 (95 % CI 0.03–0.11) [191]. Similar test performance characteristics (sensitivity 87 % [95 % CI 62–96 %] and specificity 94 % [95 % CI 88–97 %) for POCUS have been reported for Acute Chest Syndrome in children with Sickle Cell Disease [192]. Randomized controlled trial evidence suggests that lung ultrasound may be a feasible and safe substitute to chest radiography when evaluating children for pneumonia [193], which may benefit limited resource settings [194].
  Bronchiolitis/viral pneumonia: Lung ultrasound findings for bronchiolitis/viral pneumonia have been described and characterized in children. In studies performed in children with a diagnosis of bronchiolitis or viral pneumonia, ultrasound findings consisted of small sub-pleural consolidations (typically 0.25 cm in depth) with associated pleural line abnormalities, single or confluent B lines [6, 195, 196]. In general, the presence of more sonographic findings in the lungs correlated with more severe bronchiolitis/viral pneumonia [195], and the need for supplemental oxygen [6]. These ultrasound findings were helpful in distinguishing bacterial versus viral pneumonia during the H1N1 influenza A epidemic in 2009 with high inter-observer agreement [196].
  Pleural effusions: chest CT may be replaced by a POCUS with or without chest radiography in evaluating complex effusions/empyema [197].
  Pneumothorax: ultrasound has been shown to be highly accurate to diagnose pneumothoraces in the neonatal ICU in 2 prospective observational studies [198, 199]. Ultrasound has also been reported to assist with needle aspiration of a spontaneous pneumothorax in a preterm infant [200] and in a series of pediatric ED patients by tracking the sonographic “lung point” [106, 201].
  Other pulmonary pathologies: There are reports on the use of ultrasound in children to diagnose or distinguish among different pulmonary pathologies. These range from conditions such as respiratory distress syndrome (RDS) and transient tachypnea of the newborn (TTN) seen in the neonatal ICU [202], to others such as pulmonary contusions from trauma and chemical pneumonitis [203, 204], as well as different causes of wheezing in children [205].
4. Outstanding questions to be answered/voids in the literature to date
  To date, no studies have evaluated if POCUS can be used to improve antibiotic stewardship when evaluating for pneumonia.
  Serial lung POCUS evaluation may have a role in the management of ventilator-supported critically ill children and neonates.
  Additional studies are needed to investigate the role of ultrasound in administration of surfactant and respiratory support in pre-term neonates, differentiating between lower respiratory tract infections, as well as causes of wheezing in infants and children.

#### Curriculum objectives

1. Describe the* indications* for lung POCUS
  Pediatric trauma patients to assess for the presence of pneumothorax and hemothorax.
  Patients presenting with respiratory symptoms to evaluate for pneumonia versus bronchiolitis/viral pneumonia and the presence of pleural effusion versus empyema.
  Premature or full-term infants presenting with respiratory distress to assess for RDS and/or TTN [202].
2. Describe the* limitations* of lung POCUS
  Lung ultrasound may not recognize centrally located pneumonias that do not abut the chest wall (1.5 % of cases) [184, 189].
3. Describe the* relevant anatomy* to be identified with lung POCUS
  For a complete lung examination, each lung should be scanned in the longitudinal and transverse orientation in the mid-clavicular line anteriorly and posteriorly, and the mid-axillary line for a total of six scanning zones. One should scan superiorly and inferiorly from apices/clavicles to the diaphragm with liver on right, and the diaphragm and stomach or spleen on left are visualized [186, 189].
  Different relevant lung ultrasound characteristics should be identified such as A lines, B lines, confluent B lines, lung sliding, and lung consolidations with air bronchograms and small subpleural consolidations (0.25 cm with no air bronchograms).
  M-Mode may be used to confirm the presence or absence of lung sliding and a diagnosis of pneumothorax [175, 176].
4. Recognize specific* pitfalls* involved in lung POCUS
  Pitfalls to avoid when evaluating the lung include the following:
  Care must be taken to identify the left diaphragm, as the combination of spleen and air in stomach may be mistaken for pneumonia (lung consolidation with air bronchograms) [189].
  Mistaking pneumonia for thymus—thymus may appear as a lung consolidation on ultrasound but the absence of air bronchograms should differentiate tissue from pneumonia [189].
  Turn image processing features off (e.g., tissue harmonic imaging, multi-beam imaging) when assessing for lung sliding or B lines.

*Discussion of ultrasound-guided thoracentesis can be found in the procedures section. 

### Ultrasound evaluation of musculoskeletal pathology

#### Evidence

1. Summary/brief explanation of indication
  In the setting of trauma, POCUS can be used to evaluate for fractures or soft tissue injuries. It also may be used to assess proper bone alignment after fracture reduction.
  Joints can be visualized to assess for effusion, as well as to determine the best approach for and to guide arthrocentesis.
  Tendons can be visualized after trauma to evaluate for rupture or tears.
2. Relevant adult-specific literature
  Fractures:
    A study done by Marshburn et al. showed that emergency physicians with minimal training were able to identify long bone fractures in adult trauma patients with better sensitivity than clinical impression (92.9 vs. 78.6 %) [206].
    Detection of rib fractures is described in the radiology literature. Several of these studies show an increased sensitivity in detection of rib fractures by ultrasound as compared to X-ray [207, 208]. However, increased time and pain involved may outweigh the benefits of ultrasound in these settings.
    Platon et al. compared ultrasound by a radiologist to CT for identifying scaphoid fractures in patients with normal initial radiographs. Their study showed that ultrasound detected 12 out of 13 (92 %) scaphoid fractures that were missed on X-ray, but identified on CT [209].
  Joints:
    There are several cases reported in the literature regarding emergency physician use of POCUS to identify hip and ankle joint effusions and to guide arthrocentesis [210–213].
    There are also several descriptions of using ultrasound in the emergency setting to guide intra-articular blocks [214, 215].
  Tendons:
    A few case reports have been published describing the diagnosis of various tendon ruptures or tears using POCUS in the emergency department. Tendon injuries described include quadriceps rupture [216, 217], patellar tendon rupture [218, 219], triceps tear [220], hand injury with tendon laceration [221] and Achilles tendon rupture [222].
3. Relevant pediatric-specific literature
  Fractures:
    The best-described applications in the pediatric literature include identification of long bone fractures [223–227] and using ultrasound as a tool in fracture reduction [228–231].
    Hubner et al. [223] prospectively evaluated 163 children who underwent POCUS to evaluate for a suspected fracture. Ultrasound was most successful at identifying diaphyseal fractures of the humerus, femur, and forearm. Ultrasound was less accurate at identifying compound fractures, small-bone hand and foot fractures, and fractures close to joints.
    In a prospective study of 130 pediatric patients, emergency providers used POCUS to evaluate the elbow, when there was concern for elbow injury. POCUS was used to identify abnormally positioned posterior fat pads and lipohemarthrosis. The study found 98 % sensitivity (95 % confidence interval [CI] 88–100 %) of POCUS to identify the presence of a fracture [232].
    Weinberg et al. further reported on the accuracy of POCUS for identifying an elevated posterior fat pad as an indication of an elbow fracture. The study demonstrated a sensitivity of 80 % (95 % CI 51–95 %) and a specificity of 87 % (95 % CI 58–98 %) among emergency physicians with minimal training. In addition, none of the 212 pediatric patients reported any pain with the POCUS [226].
    Studies looking at pain in ultrasound versus radiographs have found similar if not lower pain scores associated with POCUS [233–235].
    POCUS has demonstrated accuracy when compared to radiography in identifying suspected forearm fractures [224, 235].
    Effective ultrasound-guided fracture reduction of the forearm has been described [228–231]. Ultrasound for this purpose has the potential to reduce the number of X-rays, associated patient transport times, and limit the use of fluoroscopy. One limitation found by Dubrosvsky et al. was that although POCUS identified patients whose reduction was successful using fluoroscopy as criterion standard, it overestimated the number needing further reduction [231].
    Additional fractures identified by POCUS in the pediatric literature include scaphoid [236], skull [237–239] and clavicle fractures [226, 233, 234].
  Joints:
    Two case series describe the use of POCUS to evaluate children presenting with acute onset of limp or hip pain [211, 240]. In one study, POCUS and the history and physical examination were able to appropriately differentiate toxic synovitis from septic arthritis and osteomyelitis in all five children [240].
    Vieira et al. [241] prospectively evaluated 28 patients who required hip ultrasonography as part of the emergency department evaluation. They determined the test characteristics of POCUS using ultrasounds performed in the Department of Radiology as the reference standard and found a sensitivity of 80 % (95 % CI 51, 95 %), and a specificity of 98 % (95 % CI 85, 99 %).
    Rabiner et al. [242] prospectively evaluated the use of POCUS in patients suspected of having radial head subluxation. They determined that although some patients with radial head subluxation had abnormal posterior fat pads (14 %), if the POCUS showed no evidence of effusion or lipohemarthrosis, reduction maneuvers could be safely attempted even if there was an unclear history of injury. The “J-sign” is a technique for verifying radial head subluxation and demonstrating successful reduction that was derived prospectively [243]. More research is needed to compare diagnostic performance of these two techniques.
    There is one case report describing the use of ultrasound-guided intra-articular lidocaine block to aid in shoulder reduction [244].
  Tendons:
    To date, there are no published studies of POCUS for the evaluation of tendon injuries in pediatric patients. There are publications in orthopedic and rheumatologic journals regarding radiology-performed ultrasound to evaluate the patellar [245], Achilles [246], and wrist [247] tendons.
4. Outstanding questions to be answered/voids in the literature to date
  Further prospective data collection is needed to clarify the utility of POCUS for the many potential musculoskeletal applications discussed above.
  It remains unclear if POCUS should be utilized in lieu of other testing, or whether it is best used as a tool for the clinician to decide which subsequent imaging study to obtain or how urgently to obtain other imaging modalities.
  More research is needed into the feasibility of teaching musculoskeletal POCUS to pediatric emergency physicians and exploring what is needed to achieve competence.

#### Curriculum objectives

##### Fractures

1. Describe the* indications* for POCUS for fracture evaluation
  POCUS is indicated in the setting of trauma to evaluate for fracture, or post-reduction of a fracture to confirm satisfactory alignment prior to splinting/casting.
2. Describe the *limitations* of POCUS for fracture evaluation
  Ultrasound has not been shown to be as effective as radiographs at identifying compound or small extremity bone fractures or fractures near joints.
  Although frequently described as less painful than the positioning that is required for plain radiographs, pain during exam or lack of cooperation may limit the quality or feasibility of the POCUS study.
3. Describe the* relevant anatomy* to be identified with POCUS for fracture evaluation
  Bones should be evaluated along their full length to evaluate the integrity of the cortex. A fracture will appear as a discontinuity in the cortex. In the case of a buckle fracture, this may be a small irregular bump in the cortical line.
  Cartilage and growth plates are hypoechoic and may be confused for a cortical irregularity (or fracture) on POCUS.
  Comparative ultrasound of the unaffected contralateral side is often helpful to differentiate fracture from normal anatomy.
  Each bone should be evaluated separately and in at least two planes.
4. Recognize specific* pitfalls* involved in POCUS for fracture evaluation
  Misdiagnosing a growth plate or cartilage as a fracture site. Evaluation of the unaffected contralateral side may prevent this error.
  Mistaking a skull suture line as a fracture line. Sutures will lead to fontanelles if open, or will be in predictable anatomic locations.
  Failure to perform a complete examination after identifying a single fracture. There may be an additional fracture present.
  In the transverse plane, failure to scan the entire circumference of the bone along its entire length may lead to missing small non-displaced fractures.
  For longitudinal scanning, bones should be scanned through their entire length in two orthogonal planes.

##### Joints

1. Describe the* indications* for POCUS evaluation of joints
  POCUS may be performed when a patient presents with joint pain or when there is suspicion for a joint effusion. This is especially useful in the evaluation of the pediatric hip joint in a child who presents with a limp.
  POCUS should be used to guide arthrocentesis of the hip, and may be useful for arthrocentesis of other joints.
2. Describe the* limitations* of POCUS evaluation of joints
  Pressure on the inflamed joint or movement of the joint during POCUS may cause discomfort or pain, and possibly limit images obtained in young children.
3. Describe the* relevant anatomy* to be identified with POCUS evaluation of joints
  Affected joints should be compared to contralateral asymptomatic joints. In the evaluation of the hip joint, the anterior synovial recess (between the iliofemoral ligament anteriorly and the femoral neck posteriorly) is measured [248]. An effusion will usually appear hypoechoic, and a measurement of greater than 5 mm or a greater than a 2-mm difference from the contralateral hip indicates a positive examination [241].
4. Recognize specific* pitfalls* involved in POCUS evaluation of joints
  Bilateral effusions will not allow for valid contralateral comparisons and may make the diagnosis of a joint effusion challenging.
  Anterior synovial recess measurements vary with hip position. Therefore, it is important to ensure the same positioning in both the affected and contralateral normal leg so that measurements are consistent and accurate.
  Patients may have symptomatic effusions that are smaller than the established parameters for pathology, and could lead to a false-negative exam [249].
  Transducer pressure may obliterate effusions, especially if small or superficial.

##### Tendons

1. Describe the* indications* for POCUS evaluation of tendons
  Tendon evaluation by POCUS is indicated when a patient presents with loss of function normally attributed to a given tendon (flex/extend), penetrating trauma in the region of tendon path, or other signs concerning for a tendon injury.
2. Describe the* limitations* of POCUS evaluation of tendons
  Location, particularly the depth of various tendons may limit the ability to use POCUS as a diagnostic tool.
3. Describe the* relevant anatomy* to be identified with POCUS evaluation of tendons
  The tendon should be evaluated along its length to look for discontinuity, retraction, thickening, thinning, changes in the architecture or effusions around the tendon.
  Tendons should be evaluated in longitudinal and transverse views. The contralateral side should be evaluated for comparison.
  It is important to be aware of the sonographic difference between tendons with and without sheaths. Normal tendons with sheaths (wrist and ankle tendons) will appear hyperechoic with a 1–2 mm hypoechoic rim of sheath. Tendons without sheaths (Achilles, patellar) are hyperechoic without the hypoechoic encasing [249].
4. Recognize specific* pitfalls* involved in POCUS evaluation of tendons
  A hematoma or edema may distort the sonographic view of a tendon.
  Knowledge of whether the tendon being imaged is covered by a synovial sheath or dense connective tissue is important to evaluate for tendonitis, tear or rupture. (i.e., in tendonitis, those surrounded by a synovial sheath will have fluid within the sheath, causing a hypoechoic rim around the tendon greater than 2 mm. Those without sheaths will manifest tendonitis with tendon thickening. With a partial tear of sheathed tendons, ultrasound may have anechoic clefts in the tendon and effusion in the sheath in contrast to tendons without sheaths, which will appear as a hypoechoic defect within the tendon [248].
  Healthy tendons display anisotropy: tendon fibers appear anechoic if the incident ultrasound beam is not perpendicular to the long axis of the fibers. At sites of injury, tendons lose their characteristic anisotropy.

*Discussion of ultrasound-guided arthrocentesis can be found in the procedures section. 

### Ultrasound evaluation of the female pelvis

#### Evidence

1. Summary/brief explanation of indications
  The female reproductive organs can be visualized sonographically in non-pregnant pediatric females who present with abdominal pain for the evaluation of ovarian cysts, pelvic inflammatory disorder (PID)/tubo-ovarian abscess (TOA), and ovarian torsion.
2. Relevant adult-specific literature
  Pelvic inflammatory disorder (PID)/tubo-ovarian abscess (TOA):
    A prospective study demonstrated that a POCUS bimanual examination performed with an endovaginal transducer in place of the internal examining hand (i.e., the sonographic bimanual examination,) was able to provide higher confidence in clinical assessment of uterine and adnexal tenderness than traditional digital bimanual examination in patients, regardless of body mass index (BMI) [250].
    Adhikari et al. demonstrated that POCUS was able to diagnose TOA in non-pregnant patients who presented with pelvic pain [251]. Of the 20 study patients, ranging from 14 to 45 years old, 14 (70 %) patients had a complex adnexal mass, 5 (25 %) patients had echogenic fluid in the cul-de-sac, and 3 (15 %) patients had pyosalpinx identified on POCUS.
    TOAs have been traditionally diagnosed on pelvic ultrasounds performed in the department of radiology, with a sensitivity of 93 % and specificity of 98 % [252]. POCUS has been shown to be less sensitive (56–93 %) and specific (83–98 %) in the diagnosis, suggesting that POCUS evaluation of TOA should be used judiciously [253].
  Ovarian torsion:
    There are case reports of emergency physicians diagnosing ovarian torsion using POCUS [254, 255].
3. Relevant pediatric-specific literature
  There is currently no literature specifically evaluating the utility of POCUS in the evaluation of ovarian cysts, pelvic inflammatory disorder or ovarian torsion in the pediatric population. However, data may be extrapolated from the studies conducted in adult patients, as many of them included adolescents in the study population, which represents the majority of the population affected by these conditions [251, 253].
4. Outstanding questions to be answered/voids in the literature to date
  As noted, there are no published data on the use of POCUS for assessing ovarian cysts, PID, TOA, and ovarian torsion among children. The majority of the data are drawn from the adult population. There are also limited data on how pelvic POCUS might be incorporated into medical decision making. Currently, there are no guidelines or protocols.

#### Curriculum objectives

1. Describe the* indications* for the gynecologic POCUS examination
  When ovarian or other gynecologic pathology is suspected such as in a female with lower abdominal pain, vaginal bleeding, or discharge.
  A ruptured ectopic pregnancy should be strongly suspected in a post-menarchal females with hypotension, syncope, and a positive pregnancy test.
2. Describe the* limitations* of the gynecologic POCUS examination
  Operator ability in consistently identifying normal ovaries, and in identifying and diagnosing other pelvic masses within the pelvis, particularly in patients with a retroverted uterus.
  Limited ability to rule out the diagnosis of ovarian torsion by POCUS, due to the fact that blood flow in the involved ovary does not exclude the diagnosis of ovarian torsion. It is difficult to demonstrate both venous and arterial waveforms in all cases and torsion may be intermittent, demonstrating variability in detectable blood flow on color Doppler.
3. Describe the* relevant anatomy* to be identified with the gynecologic POCUS examination
  Pediatric gynecology can be evaluated either in a trans-abdominal, transperineal/translabial, or trans-vaginal approach. However, the trans-vaginal approach should be avoided in pre-coital, pre-pubescent females.
  The bladder appears as a well-circumscribed, rectangular, fluid-filled, anechoic structure in the transverse plane. The cervix can be visualized just posterior to the bladder with the body of the uterus and uterine stripe cephalad and the vaginal stripe caudal to the cervix. The uterus appears as an isoechoic structure with thick walls and a well-defined border. Retained mucous secretions prior to ovulation may make the endometrium appear heterogeneously echogenic and should not be confused with intrauterine tumors. If an intrauterine device (IUD) is in place, this will typically appear as a hyperechoic linear structure with reverberation artifact. An appropriate location within the body of the uterus, not perforating the myometrium, should be confirmed. The posterior cul-de-sac, or Pouch of Douglas, is posterior to the uterus and may be the site of free fluid collection. A small amount of physiologic free fluid is acceptable, but clinical correlation is advised. Ovaries appear as isoechoic to hypoechoic structures containing multiple anechoic follicles, giving an appearance similar to that of a ‘chocolate chip cookie.’
  Ovarian cysts: Simple cysts of less than a centimeter in diameter are defined as ovarian follicles. Simple cysts should be anechoic, smooth walled structures with posterior acoustic enhancement. More complex appearances may be due to hemorrhagic cysts, which may have varying appearances depending on the stage of the clot. Complex, multiloculated cysts require further evaluation with follow-up US or MRI.
  Pelvic inflammatory disorder (PID)/tubo-ovarian abscess (TOA): The normal fallopian tube is not always visible on ultrasound. PID can present with a thickened tubular adnexal structure that is representative of an inflamed fallopian tube. Edematous walls and endosalpingeal folds have been described as the ‘cogwheel sign.’ A blocked fallopian tube may become dilated and fill with fluid, showing a hydrosalpinx or pyosalpinx. A TOA may cause loss of the architectural definition of the adnexa, with multiple thick-walled, dilated fluid-filled structures that contain fluid-debris and air-fluid levels.
  Ovarian torsion: A torsed ovary will often appear enlarged, amorphous and hypoechoic due to edema from obstruction of lymphatic and venous drainage. Free fluid may be present in the pelvis. Doppler evaluation of the ovary and any adjacent masses should be performed. The whirlpool sign is a dynamic sign demonstrated by placing the ultrasound transducer at right angles to the axis of the ovarian pedicle and fanning it to and fro. The whirlpool sign appears as “a clockwise or counterclockwise wrapping of the hypoechoic ovarian vessels around the central axis” of the pedicle [256]. The ovaries have a dual blood supply and many articles have described normal Doppler flow with torsion [257, 258]. However, studies consistently reveal that venous flow is lost before arterial flow; therefore, optimizing power Doppler settings to detect venous flow significantly enhances the ability to identify partial or early torsion.
4. Recognize specific* pitfalls* with the gynecologic POCUS evaluation
  Failure to ensure a full bladder for use as an acoustic window in the transabdominal view and an empty bladder for the endovaginal examination.
  Failure to recognize an enlarged ovary as ovarian torsion, until proven otherwise, despite the presence of blood flow.
  Misidentifying uterine vasculature as a thin-walled hydrosalpinx on cross-sectional image planes. Doppler evaluation can usually resolve this issue.
  Misidentifying bowel loops for ovarian or other adnexal structures.
  Failure to identify abnormal adnexal masses and/or significant pathologic ovarian abnormalities

### Ultrasound evaluation of the pelvis in the first trimester of pregnancy

#### Evidence

1. Summary/brief explanation of indications
  The female reproductive organs can be sonographically visualized in adolescent females who present with abdominal pain or vaginal bleeding in their first trimester of pregnancy. The primary indication for clinician-performed sonographic evaluation of the female pelvis in the first-trimester pregnancy is in the identification of an intrauterine pregnancy; thereby, effectively excluding ectopic pregnancy in the vast majority of patients. There are some defined groups of patients at high risk for ectopic pregnancy in whom this approach is not adequate to exclude ectopic/heterotopic pregnancy. POCUS in the evaluation of first-trimester pregnancy can also be used to determine fetal heart rate, gestational age, and identification of abdominal free fluid. With higher levels of training and experience, point-of-care ultrasound may also assist in the diagnosis of intrauterine demise, anembryonic gestation, gestational trophoblastic disease, various abortion states (inevitable, incomplete, complete, etc.) and adnexal pathology.
2. Relevant adult-specific literature
  IUP:
    Jang et al. assessed the learning curve of emergency physicians for first-trimester pregnancy POCUS and found that the sensitivity and specificity for identifying an IUP were 80 and 86 %, respectively, for their first 10 examinations, and increased to 100 % sensitivity and specificity after performing 40 examinations [259].
    McRae et al. conducted a systematic review which demonstrated that POCUS was able to identify a definitive intrauterine pregnancy with a sensitivity of 90 % and a specificity of 98 % in women presenting with pelvic pain and/or bleeding during their first trimester of pregnancy [260].
    In patients in whom an IUP was determined by bedside ultrasound, the ED length of stay was 21–28 % shorter than when ultrasound was performed by the department of radiology [261]. In a more recent study, patients who received bedside ultrasonography for complications of first-trimester pregnancy had more than 2 h of time savings in average length of stay whether they received transabdominal ultrasound (2.8 h lower length of stay) or transvaginal ultrasound (2.2 h no length of stay). In both cases, this represented a greater than 35 % decrease in length of stay for patients receiving POCUS [262].
  Ectopic pregnancy:
    A meta-analysis by Stein et al. demonstrated that emergency physicians were able to screen for an ectopic pregnancy with POCUS with a sensitivity of 99.3 %, negative predictive value of 99.96 % and a negative likelihood ratio of 0.08 across a wide variety of practice environments [263].
    Moore et al. found that in patients with suspected ectopic pregnancies on pelvic POCUS, free intraperitoneal fluid found in Morison’s pouch could be rapidly identified by POCUS and was predictive of the need for operative management, with a positive likelihood ratio of 112 [264].
    Tayal et al. found that earlier identification of an indeterminate POCUS pelvic ultrasound with the eventual diagnosis of an ectopic pregnancy had a higher rate of medical methotrexate treatment and a reduced rate of invasive surgical treatment (36 vs. 83 %) compared with ectopic pregnancy patients diagnosed at initial ED visit [265]. The generally accepted “discriminatory zone”, or minimal level of β-HCG at which the first sign of an intrauterine pregnancy (a double decidual sac) can be sonographically visualized, is 1500 mIU/ml. However, many ectopic pregnancies present with β-HCG levels less than 1500 mIU/ml, and many of these have sonographic evidence of ectopic pregnancy [266–269]. Furthermore, many point-of-care sonologists consider the double decidual sac insufficiently specific to rule in intrauterine pregnancy definitively, and the discriminatory zone *cannot* be extrapolated to expected levels at which any of the more definitive sonographic signs of IUP such as yolk sac, fetal pole, or cardiac motion should be seen. These considerations argue that POCUS pelvic ultrasounds should be performed regardless of β-HCG levels in any pregnant patient with a suspicion for ectopic pregnancy.
    Heterotopic pregnancy is much more prevalent in women who have received ovarian induction therapy, and may be diagnosed by POCUS [270]. Although the primary focus of clinician-performed ultrasonography in first-trimester pregnancy is the identification of intrauterine gestation, it is prudent to evaluate the adnexa for signs of heterotopic pregnancy even after an IUP has been identified. Women who have received assistive reproductive therapies should always be evaluated for the presence of ectopic or heterotopic pregnancy even when an IUP is identified. Since identification of extrauterine pregnancy is not a primary indication of the POCUS evaluation, this examination should usually be performed by imaging specialists.
    When POCUS does not identify a definitive IUP (absence of gestational sac with a yolk sac or fetal pole), timely follow-up by gynecologists and/or further ultrasonography by imaging specialists will be required. If the POCUS is suggestive of an ectopic pregnancy (tubal ring, adnexal mass, pseudogestational sac, free fluid in the abdomen, endometrial mantle measuring less than 5–8 mm concerning for an interstitial pregnancy), patients should undergo urgent ultrasonography by imaging specialists and gynecology consultation [271, 272].
    The use of POCUS has demonstrated decreased time to surgery for ectopic pregnancies, shortened lengths of ED stay for patients with normal pregnancies, and decreased number of return ED visits with a subsequent ruptured ectopic [260, 273].
3. Relevant pediatric-specific literature
  The use of POCUS in pregnant pediatric patients between the ages of 13–21 years old reduced ED length of stay from 149 min (range 7–506 min) to 82 min (range 1–901 min) (*p* < 0.001) when an intrauterine pregnancy was identified on POCUS as compared to when ultrasound was performed by the department of radiology [274].
  There have been limited studies to date specifically evaluating the application of POCUS in adolescents who present to a pediatric ED with first-trimester pregnancy complaints to differentiate between an IUP and an ectopic pregnancy as well as to diagnose a threatened abortion. However, with further training and credentialing, it is likely that pediatric point-of-care sonologists will attain similar accuracy in identifying and managing first-trimester pregnancy as their colleagues in the adult ED.
4. Outstanding questions to be answered/voids in the literature to date
  Published data on the use of POCUS in the pediatric ED in assessing adolescents with first-trimester pregnancy are limited.
  There are no data in children, and limited data in adults regarding the use of POCUS as an adjunct in the evaluation of other common emergency gynecological conditions, such as pelvic inflammatory disease and ovarian torsion.

#### Curriculum objectives

1. Describe the* indications* for POCUS in first-trimester pregnancy
  Indications for first-trimester POCUS include evaluation for IUP, identification of yolk sac, fetal pole, detection of fetal heart rate, determination of gestational age, and identification of abdominal free fluid. Understand the principle of exclusion of ectopic pregnancy by identification of IUP.
2. Describe the* limitations* for POCUS in first-trimester pregnancy
  Limitations include the ability to rule out ectopic pregnancy when ultrasound does not show definitive evidence of an IUP. In this case, an ‘indeterminate’ exam revealing an empty uterus or a nonspecific intrauterine sac or endometrial fluid collection/echogenic material requires correlation with the patient’s quantitative beta hCG and other clinical findings.
  Patients who have undergone assisted reproductive technology are at high risk for heterotopic pregnancies, and, therefore, require an ultrasound examination by the imaging specialists to rule out the presence of an ectopic pregnancy.
  POCUS is not sufficient for identifying or ruling out fetal anomalies or for evaluating pelvic anatomy after vaginal-rectal surgery.
3. Describe the* relevant anatomy*/findings to be identified with POCUS in first-trimester pregnancy
  First-trimester pregnancy should be evaluated in a transabdominal and/or transvaginal approach, depending on clinician training, the availability of equipment, and the gestational age of the fetus
    Transvaginal ultrasound can identify an intrauterine pregnancy at approximately 5 weeks gestation, and transabdominal ultrasound can identify an intrauterine pregnancy at approximately 6–7 weeks gestation.
  The first sonographic confirmation of an IUP is the yolk sac at 5–6 weeks, followed by an embryo with cardiac activity at 6 weeks, and finally a fetal pole at approximately 7 weeks. An intradecidual sac can be seen at 4–5 weeks, followed by a double decidual sac at 5 weeks; however, neither of these definitively confirms the diagnosis of an IUP.
  If the pregnancy is intrauterine, the endomyometrial mantle should be measured. An endomyometrial mantle thickness of at least 5–8 mm is considered normal and minimizes the risk of an interstitial or cornual ectopic pregnancy.
  In most cases, transabdominal sonography is facilitated by the presence of a full bladder. Conversely, transvaginal ultrasonography is generally easier with empty bladder. The bladder should be identified as a well-circumscribed, fluid-filled, anechoic structure. The uterus appears as a hypoechoic structure with thick walls and a well-defined border. In a typical anteroverted and anteroflexed uterus, the cervix appears just posterior to the bladder angle with the uterine body superior to the bladder and the vaginal stripe immediately posterior to the bladder in the sagittal view. The rectouterine cul-de-sac, or Pouch of Douglas, is posterior to the cervix and may be the site of free fluid collection. The ovaries may be visualized lateral to the uterus on either side and appear as isoechoic to hypoechoic structures containing multiple anechoic follicles. The ovaries should be identified in two orthogonal planes, where possible. This may be limited by the patient’s habitus, bowel gas, pelvic anatomy, sonologist skill, or the quality of the ultrasound machine.
  Depending on the sonographic size, certain presets on the ultrasound machine may allow for gestational dating based on crown-rump length.
  If cardiac activity is noted, M-Mode can be used to measure the fetal heart rate. Doppler should not be used in the first trimester to determine fetal heart rate, as this carries a theoretical risk of exposing the fetus to excessive levels of ultrasound energy with the potential for fetal damage and birth defects.
  In addition to evaluating for an intrauterine pregnancy, the pelvis should be interrogated for evidence of free fluid.
  In unstable patients, the right upper quadrant (Morison’s pouch) should also be evaluated as free fluid in this area in a pregnant female should be considered as a ruptured ectopic pregnancy with significant hemorrhage until proven otherwise.
4. Recognize specific* pitfalls* involved in performing POCUS in first-trimester pregnancy
  Attributing an empty uterus to a very early IUP, an ectopic pregnancy, or a completed spontaneous abortion (all of these entities are possible in the “indeterminate exam”).
  Mistaking a nonspecific intrauterine sac or a pseudogestational sac for a gestational sac.
  Incorrectly identifying a pregnancy as within the uterus, when in fact it is outside the uterus or interstitial.
  Failure to consider or to identify heterotopic pregnancy.
  Misidentifying an adnexal mass for an ectopic pregnancy.
  Failure to identify an embryonic demise, or diagnosing embryonic demise in a live IUP.
  Failure to perform a POCUS study when the βHCG is below 1500 mIU/L

### Ultrasound evaluation of pyloric stenosis

#### Evidence

1. Summary/brief explanation of indication
  The pylorus can be evaluated sonographically in infants in whom there is clinical concern for idiopathic hypertrophic pyloric stenosis (HPS).
  The typical history includes non-bilious, projectile vomiting in a one-month old infant shortly after feeding. The classic physical exam finding of a palpable “olive” and the laboratory findings of hypochloremic hypokalemic metabolic alkalosis are not commonly seen [275–279].
2. Relevant pediatric-specific literature
  Ultrasound has been utilized by radiologists to diagnose HPS since 1977 and is now the primary modality used to diagnose HPS [280].
  Recent literature describes the ability of both emergency physicians [281] and resident surgeons [282, 283] to accurately diagnose HPS by bedside ultrasonography.
  A prospective study of PEM fellows and a PEM attending physician found 100 % (95 % CI 62–100 %) sensitivity and 100 % (95 % CI 92–100 %) specificity when evaluating patients with suspected HPS, with measurements of pyloric muscle wall width and length that were not statistically different (*p* = 0.50 and *p* = 0.79, respectively) from those of Radiology specialists [284].
3. Outstanding questions to be answered/voids in the literature
  Literature from the emergency medicine and surgery publications describes either small case series, or high probability patients. The test characteristics of POCUS in the evaluation of the pylorus for HPS require continued evaluation.
  Variable measurements have been used in radiology texts to establish the diagnosis for HPS [285–289]. There is currently no definitive consensus regarding absolute measurements.

#### Curriculum objectives

1. Describe the* indications* for sonography for HPS
  The sonographic evaluation for pyloric stenosis should be considered in young infants with non-bilious emesis.
2. Describe the* limitations* of the pyloric examination
  Difficulty with pyloric visualization may arise from gastric over-distention, which may displace the pylorus posteriorly.
  Gastric air or bowel gas may cause shadowing artifact over the area of interest.
3. Describe the* relevant anatomy* to be identified in the examination of the pylorus
  The pyloric muscle is in continuity with the gastric wall and arises to the right of midline. Using the liver as a sonographic window, the gastric wall is identified overlaying a gas and/or fluid-filled stomach. The gastric wall is then traced caudally to the right of midline along the lesser curvature of the stomach until it meets the pyloric antrum.
  The beginning of the pyloric antrum is identified by the incisura angularis, which appears like a notch in the gastric wall’s serosal surface.
  The distal end of the pylorus is identified by the interface between the pyloric sphincter and the first portion of the duodenum or duodenal cap. The appearance is dependent on whether the pyloric sphincter and/or channel muscles are relaxed or contracted. However, the thicker muscle wall of the pylorus is easily recognized in contrast to the thinner-walled duodenum. Another notch in the wall, the duodenal pyloric constriction, may also be noted at this junction.
  Diagnostic measurements include the pyloric muscle thickness and the pyloric channel length. A pyloric muscle thickness of less than 2 mm is considered normal, between 2 and 2.9 mm seen in both normal and pylorospasm, and greater than 3 mm considered diagnostic of HPS [285, 286, 290, 291]. The channel length may be difficult to measure in a normal patient and is less consistent than the more easily appreciated pyloric muscle thickness. The channel length is considered abnormal if it is greater than 15 mm [290, 292–294]. Both measurements should occur in tandem, and caution would be prudent if only a thickness or length measurement was abnormal.
  A normal pylorus allows gastric contents to pass through the relaxed pyloric canal with gastric peristalsis. The normal pylorus may also be transiently thickened, but returns to normal thickness after muscular contraction in contrast to HPS, where the pylorus is continuously thickened. These observations may require viewing the pylorus for 5–10 min. Wall relaxation and distention with passage of gastric contents are not seen with pyloric stenosis.
  In addition to the abnormal measurements, other sonographic signs seen in HPS include the “antral nipple” sign seen in long-axis view where the opposed mucosa layers project back into the antrum, the “shoulder” sign, created by the circular pyloric muscle similarly projecting into the antrum, and the “donut” or “target” sign seen when the pylorus is viewed in cross section. In addition, there is failure of gastric contents to pass through the pyloric channel when visualized in real time.
4. Recognize specific* pitfalls* involved in POCUS of the pylorus.
  Pitfalls to avoid when performing the pyloric examination include the following:
  Pylorospasm and/or normal pyloric peristaltic contraction may mimic HPS findings. With borderline measurements, the area should be observed for 5–10 min to observe for pyloric relaxation and passage of gastric contents (thereby excluding HPS).
  Misidentification of gastric or duodenal wall for the pyloric wall may result in a false-negative exam.
  Tangential measurements may erroneously exaggerate pyloric thickness.
  Non-visualization of the pylorus secondary to gastric air, or a posterior position from gastric distention may prevent an effective evaluation. Positioning the patient in a right or left decubitus oblique position (respectively) may facilitate imaging.
  A struggling or crying infant makes examination much more challenging. Gel should be at ambient temperature and a pacifier dipped in sucrose-water solution may be helpful. The patient may be examined in the arms of family

### Ultrasound evaluation of the soft tissue

#### Evidence

1. Summary/brief explanation of indications
  Differentiating between cellulitis and abscess can be difficult based on clinical examination alone, as there are overlapping features of both. Soft tissue POCUS can be utilized to identify the presence, location, and size of an abscess.
  POCUS can localize soft tissue foreign bodies. This is of particular utility if there is concern for a radiolucent object.
2. Relevant adult-specific literature
*Skin and soft tissue infections* POCUS in adult patients is more accurate than clinical examination alone. One study demonstrated clinical examination sensitivity for identifying an abscess of 86 % (95 % CI 76, 93 %), specificity of 70 % (95 % CI 55, 82 %). Sensitivity when POCUS was added to the clinical examination was 98 % (95 % CI 93, 100 %), and specificity was 88 % (95 % CI 76, 96 %) [295].
  POCUS can be performed with accuracy even among novice sonographers. The accuracy of novice sonographer identification of abscess by POCUS had a sensitivity of 97 % (95 % CI 83, 100 %) and a specificity of 67 % (95 % CI 24, 94 %) as compared to clinical exam which had a sensitivity of 76 % (95 % CI 58, 89 %) and a specificity of 83 % (95 % CI 36, 99 %) [296].
  POCUS can help guide management interventions in cases of skin soft tissue infections. In one study, ultrasound altered physician intervention in 50 % of cases. The addition of ultrasound either assisted with identification of clinically occult abscesses or helped avoid invasive procedures in cases of cellulitis without abscess [297].
  A recent pilot study described a clinical decision rule for ultrasound diagnosis of Methicillin Resistant Staphylococcus Aureus (MRSA) abscesses. The findings of a lack of a well-defined abscess edge, small volume and an irregular shape together were seven times more likely to be culture positive for MRSA. The decision rule demonstrated a sensitivity of 89 % (95 % CI 85, 93 %) and specificity of 44 % (95 % CI 41, 48 %) [298].
*Soft tissue foreign bodies* The authors have cited wide ranges in diagnostic accuracy for detection of foreign bodies in simulation-based studies. For example, chicken thighs, beef cubes and cadaveric models have been used, with sensitivities for localization ranging from 43–98 % and specificities ranging from 59–98 % [299–301].
  In several case series of radiology ultrasound and POCUS, ultrasound was able to correctly identify the presence of a retained foreign body [302–305].
3. Relevant pediatric-specific literature
*Skin and soft tissue infections* Clinical examination has been shown to be an unreliable method of distinguishing cellulitis from abscess requiring drainage among pediatric EM physicians [306, 307]. One prospective pediatric study found POCUS to be 90 % sensitive (95 % CI 77, 100 %) and 83 % specific (95 % CI 66, 94 %) for distinguishing abscess from cellulitis, as compared to clinical suspicion, which had a sensitivity of 75 % (95 % CI 70, 97 %) and a specificity of 80 % (95 % CI 66, 94 %) [308]. In that study, POCUS resulted in a change in management in 22 % of cases.
  Another prospective study of 65 children with clinical examination findings consistent with a skin and soft tissue infection sought to compare the test characteristics of clinical examination to those with the addition of POCUS. In this study, the sensitivity of clinical examination for the detection of abscess was 78 % (95 % CI 71, 84 %) with a specificity of 66 % (95 % CI 47, 81 %). The addition of POCUS resulted in statistically significant improvement in sensitivity to 97 % (95 % CI 90, 99 %) but not of specificity (69 %; 95 % CI 57, 72 %) [309].
  A third study demonstrated that POCUS did not improve upon the already optimal test characteristics of clinical examination alone when physicians were certain of their diagnosis; however, POCUS may be of use in cases of clinical uncertainty [310].
  Soft tissue POCUS is a skill that is easily mastered after even a short period of instruction. One study demonstrated that after a 6-h instructional period, novice pediatric emergency medicine fellows and attendings were able to perform adequate scans and their interpretations agreed with an expert sonologist (kappa 0.8) [311].
*Soft tissue foreign bodies* A pediatric study of 131 wounds compared the diagnostic accuracy of radiographs to POCUS alone, and to ultrasound plus patient perception of a tissue foreign body [312]. There was no statistically significant difference in the diagnostic accuracy of radiographs as compared to ultrasound alone for the diagnosis of tissue foreign body. However, the specificity of POCUS when combined with patient perception [76.5 % (95 % CI 66.9, 84.5)] was lower than that of radiography [88 % (95 % CI 80.8, 94.3)].
  One pediatric case report described how easily POCUS can be incorporated into the clinical evaluation of wounds that have a high probability of tissue foreign body. In this report, a wood splinter was clearly visualized under ultrasound and ultrasound further assisted in guiding foreign body removal [313].
4. Voids in the literature
*Skin and soft tissue infections* There are no published reports regarding the impact that POCUS has on emergency department outcome measures (including morbidity, length of stay, cosmetic results, and cost effectiveness).
  To date, no studies have looked at emergency provider performed ultrasound for the management of soft tissue neck masses.
*Soft tissue foreign bodies* There is a paucity of pediatric literature evaluating ultrasound for the diagnosis and management of foreign bodies.
  There have been no published pediatric prospective studies comparing outcomes of foreign body removal using ultrasound guidance compared to blind removal after radiographic or sonographic localization.
  Currently, there are no published reports on ultrasound training for pediatric emergency providers for localization of foreign bodies either in vivo or with a simulation-based design.

#### Curriculum objectives

1. Describe the* indications* for soft tissue POCUS
  Indications for skin and soft tissue ultrasound include:
  Suspicion for a soft tissue infection or lymphadenitis such as the presence of a tender, fluctuant, erythematous lesion.
  Concern for a retained foreign body such as with a history of a soft tissue infection not improving with appropriate treatment, or a history of trauma with the potential for foreign body exposure.
2. Describe the* limitations* of soft issue POCUS
  Ultrasound cannot differentiate between infectious and inflammatory fluid collections [314].
  The development of an abscess in an area of cellulitis can make the distinction between abscess with cellulitis and cellulitis alone challenging [315].
  In the evaluation of tissue foreign body, any introduction of air, whether from the trauma itself, or from the manipulation of the wound, can obscure the identification of the foreign body on ultrasound or prompt the misdiagnosis of a foreign body when there is none actually there [315].
3. Describe the* relevant anatomy* to be identified with soft tissue POCUS
  Normal soft tissue architecture consists of layers of epidermis and dermis overlying muscle, tendons, and ligaments. Vessels and nerves may also be seen. The most important anatomic landmark for evaluation of soft tissue infections and foreign bodies is the superficial muscle fascia (or tendon or bone in areas without muscle). This distinguishes superficial processes from deeper ones which require complex management.
  “Cobblestoning” (lobular appearance of the subcutaneous fat) is the classic sonographic finding with cellulitis, and is secondary to the interstitial edema within the tissue.
  An anechoic (dark) fluid-filled irregularly shaped lesion with posterior acoustic enhancement is consistent with an abscess. Gentle downward pressure on the lesion may demonstrate internal movement of pus. Color Doppler can be used to confirm that the lesion is not a vessel or lymph node.
  A loculated abscess will have internal echoes and septations within the fluid-filled space.
  A lymph node will appear as well-circumscribed structure with a hypoechoic cortex and hyperechoic medulla. With color Doppler, internal flow will be seen, and there may be surrounding hyperemia.
  Both radiopaque and radiolucent foreign bodies appear hyperechoic on ultrasound, often with posterior acoustic shadowing which may assist in the detection of the foreign body [306–310]. Other sonographic findings include reverberation (or “comet tails”) artifact, and a hypoechoic halo. The latter is caused by an inflammatory reaction, which will be more common with organic foreign bodies, and those that are less acute (present for more than 24–48 h).
4. Recognize specific* pitfalls* involved with soft tissue POCUS
  Mistaking a vessel, hematoma, or lymph node for an abscess.
  Mistaking subcutaneous edema for a drainable fluid collection.
  Failure to increase the depth appropriately, such that a deep tissue abscess is not recognized. Conversely, a superficial abscess can be missed if there is insufficient gel or if the depth is too great [315].
  While an abscess often appears anechoic or hypoechoic, it can also appear as an isoechoic or hyperechoic collection. Optimal gain settings, recognition of posterior acoustic enhancement, and identification of the to-and-fro motion of pus with probe pressure may be needed to detect such abscesses.
  Small and deep foreign bodies can be challenging to identify.
  Foreign bodies may only be identifiable by the reverberation and/or shadowing artifacts that they cause.
  A foreign body can easily be confused with normal soft tissue structures, such as fibrous septations, muscle fascia, bones, and cartilage, especially in the hands and feet. As with foreign bodies, these structures appear hyperechoic and may cause shadowing [308].
  Care must be taken to scan through entire tissue area systematically and in two orthogonal planes so as not to miss the presence of a foreign body.
  When using color Doppler, appropriate attention to color flow gain and scale is necessary.

*Discussion of ultrasound-guided abscess drainage can be found in the procedures section. 

### Ultrasound evaluation of the testicles

#### Evidence

1. Summary/brief explanation of indication
  The evaluation of acute testicular pain is a time-sensitive clinical challenge requiring accurate diagnosis.
  POCUS is indicated for patients with clinical findings suggestive of an acute process that require immediate management (i.e., testicular torsion, traumatic hematomas and incarcerated hernias). These need to be distinguished from less urgent entities such as epididymitis, epididymo-orchitis, and a hydrocele.
2. Relevant literature in adult patients
  In 2001, Blaivas et al. performed a retrospective study of POCUS in the evaluation of 36 ED patients presenting with acute testicular pain. To date, this is the only emergency physician-performed ultrasound study of its kind. They accurately diagnosed multiple testicular entities with a sensitivity and specificity of 95 and 94 %, respectively. All possible pathologies were included and there were no missed cases of testicular torsion [316].
  In 2013, Cannis et al. presented a case report of a 22-year-old male for which the use of POCUS facilitated early diagnosis of a ruptured testicle and allowed for prompt urological consultation and timely surgical repair [317].
  In 2009, Bomann et al. reported the use of POCUS by an emergency physician to diagnosis torsion in a 26-year old with successful detorsion [318].
3. Relevant pediatric literature
  In 2000, Blaivas et al. presented a case report of a 14-year-old boy found to have testicular torsion by a POCUS examination that led to rapid diagnosis and subsequent successful salvage of the affected testicle [319].
  There are also case reports in the literature of POCUS identification of testicular teratoma [320] and testicular dislocation secondary to trauma [321].
4. Outstanding questions to be answered/voids in the literature to date
  The use of POCUS by pediatric emergency physicians in pediatric patients has not been studied other than case reports.
  Prospective ED studies are needed to assess the impact of POCUS in pediatric patients presenting with acute scrotum.
  Research is needed into the feasibility of teaching testicular POCUS imaging to pediatric emergency physicians and exploring how to achieve and assess competence.

#### Curriculum objectives

1. Describe the* indications* for testicular POCUS
  For clinicians with expertise in POCUS evaluation of the testicle, the exam is indicated for patients with acute scrotal or inguinal pain, swelling, or trauma.
2. Describe the* limitations* of testicular POCUS
  Studies have shown that the identification of flow in the normal pre-pubertal testis may be difficult or impossible, making the diagnosis of torsion on the contralateral, symptomatic side more challenging.
  Intermittent torsion may present with relatively normal to increased flow, thus making definitive diagnosis difficult. Serial ultrasound examinations are not likely to be of benefit and the patient needs to be evaluated by urology for possible orchiopexy.
3. Describe the* relevant anatomy* to be identified with testicular POCUS
  Relevant anatomy includes the testicular skin, tunica vaginalis (with or without hydrocele), capsule (tunica albuginea), spermatic cord, testicular vein and artery, appendix testes, epididymis, and testicle.
  While performing bilateral testicular B-mode sonography, continuous clips through the entire organ in the longitudinal and transverse planes are ideal. If recording in still images, necessary views include central, medial, and lateral views in the longitudinal plane, and superior, mid, and inferior views in the transverse plane. The “raphe” view, a comparison of both testes in transverse orientation, evaluates the size, echogenicity, vascularity of each testis in a side-by-side image. Color and/or spectral Doppler are needed to evaluate flow to the testicle and epididymis. Having the patient perform a Valsalva maneuver may help identify varicoceles.
  A torsed appendix testis will appear as a 5 mm or larger, spherical or round appendage with variable echogenicity, increased periappendiceal blood flow, and no blood flow within the torsed appendage.
  Epididymitis will appear as an enlarged epididymis with variable echotexture and increased blood flow. There may be scrotal wall thickening and an associated reactive hydrocele.
  Orchitis may present sonographically only as asymmetric or bilateral hypervascularity on color Doppler. The testicle(s) may be diffusely enlarged and have variable echogenicity. A reactive hydrocele and scrotal wall edema may further support the diagnosis.
  A hydrocele will appear as an anechoic fluid collection adjacent to the testis, and is common with all testicular pathology.
  A hernia will appear as a fluid-filled structure within the inguinal canal, spermatic cord, and/or scrotal sac. Depending on whether there is incarceration/strangulation, there may be peristalsis and mucosal blood flow identified by color Doppler.
4. Recognize specific* pitfalls* involved in testicular POCUS
  Failing to use proper color flow and Doppler gain settings may lead to a false interpretation of torsion due to the low velocity flow within the intratesticular vessels.
  Mistaking extratesticular blood flow for intratesticular flow may lead to a false-negative interpretation of torsion.
  The presence of increased paratesticular flow may occur with chronic torsion, intermittent torsion, or orchitis.
  Failure to recognize the presence of detorsion. After detorsion, there may be increased testicular blood flow mimicking epididymitis or orchitis or there may be normal testicular blood flow. These patients are at high risk for retorsion and require urological evaluation.
  A scrotal pearl is a benign calcification that can be seen and is idiopathic in nature. It appears as a hyperechoic lesion with acoustic shadowing and may be mistaken for pathology.
  Relying on a diagnosis of torsion of the appendix testis, orchitis, or epididymitis before ruling out testicular torsion which can lead to false-negative exams

### Ultrasound evaluation of the urinary tract

#### Evidence

1. Summary/brief explanation of indications
  POCUS can be used to evaluate the kidneys, collecting systems, and bladder in pediatric patients to assess for hydronephrosis, the presence of renal calculi, abscesses, and cysts.
2. Relevant adult-specific literature
*Renal colic/hydronephrosis* Several studies have looked at the ability of POCUS to detect hydronephrosis in the setting of renal colic. Results show a sensitivity between 76 and 87 %, and a specificity between 78 and 83 %, using CT as the gold standard [322–325]. The absence of hydronephrosis on ultrasound predicts easier passage of calculi, thereby mitigating the clinical need for further evaluation [326]. There are varying reports on the potential for POCUS to identify the actual calculi, partly dependent on size and location of the stone, with a sensitivity between 61 and 100 %. The size of the calculi identified on ultrasound tends to be overestimated [327–329]. POCUS can be the only imaging acquired in the initial assessment of a patient with suspected nephrolithiasis, with no increase in serious adverse events, return ED visits or hospitalizations [330].
*UTI/pyelonephritis* POCUS may detect complications such as renal abscesses [331].
*Renal cysts* POCUS is helpful in diagnosing and evaluating renal cysts [332, 333].
3. Relevant pediatric-specific literature
*Renal colic/hydronephrosis* Although much less common in pediatric patients, the incidence of urolithiasis is increasing, with some accounts reporting as much as an 86 % increase in children diagnosed with renal colic in the past decade [334–336]. Ultrasound is less sensitive than CT in identifying urolithiasis, especially ureteric stones [337].
*UTI/pyelonephritis* The majority of pediatric patients diagnosed with a UTI do not require imaging in the acute setting. However, for those patients who fail to respond to standard treatment and present to the emergency department, POCUS may assist in identifying abnormalities such as renal or perirenal abscesses or hydronephrosis. Further alternative imaging may be necessary to evaluate for renal scarring or reflux, as ultrasound has been shown to be less sensitive and specific than other modalities such as DMSA in these settings [338–343].
*Renal cysts* Renal cysts are congenital or acquired. They are uncommonly seen in the pediatric population, but the incidence increases with age. Cysts may be noted especially in at-risk patients with a family history of polycystic kidney disease [344–346].
4. Outstanding questions to be answered/voids in the literature to date
  There are currently minimal data on the use of POCUS in the pediatric emergency department for assessing the urinary tract. It is limited to reports on bladder assessment, and a few emerging studies on POCUS for suspected renal colic.
  Since the test characteristics of ultrasound in the assessment of pediatric urinary tract ailments are currently unknown, it is unclear in which patients a bedside renal ultrasound should be performed, and how the results of the ultrasound should be incorporated into medical decision making.

#### Curriculum objectives

1. Describe the* indications* for POCUS of the urinary tract
  Indications for performing POCUS of the urinary tract include the following: flank pain, hematuria, dysuria and unexplained renal failure, depending on the clinical context. POCUS may be used to evaluate for hydronephrosis, the presence of renal calculi, cysts or tumors.
2. Describe the* limitations* of POCUS of the urinary tract
  Identification of obstructing ureteral calculi is beyond the skill of most point-of-care sonologists.
  Normal ureters are not visualized on ultrasound.
  Ultrasound is not useful in the diagnosis of pyelonephritis; however, it may assist in identifying underlying causes or complications.
  Ultrasound is not sensitive or specific for diagnosing renal scarring.
  The examination may be technically limited by bowel gas, abdominal pain, the patient having an empty bladder as well as large body habitus.
3. Describe the* relevant anatomy* to be identified in the POCUS examination of the urinary tract
  The urinary tract should be evaluated by visualizing both kidneys and the collecting systems, together with the bladder. Each kidney needs to be scanned in the longitudinal and transverse orientation, ensuring that the entire kidney together with the collecting system is evaluated. Each kidney is enclosed in a layer of fascia (Gerota’s fascia). Between this fascia and the fibrous renal capsule, there is a layer of perinephric fat. The kidneys themselves are divided into the outer cortex, an inner medulla and the renal sinus, which include the renal collecting system, blood supply and adipose tissue. The renal pelvis refers to the part of the collecting system between the convergence of the calyces and the transition into the proximal ureter. In healthy individuals, these cannot be seen on ultrasound. The bladder is an anechoic structure, surrounded by an echogenic wall. It should be evaluated in both the transverse (when it can appear trapezoid in shape) and longitudinal orientation. Ureteral jets can also be viewed with the application of color Doppler in the transverse orientation.
4. Recognize specific pitfalls involved in POCUS of the urinary tract.
  Pitfalls to avoid when evaluating the urinary tract include the following:
  Incorrectly ascribing hydronephrosis to obstruction in a patient with a full bladder or one who is over hydrated. Ideally patients undergoing evaluation of the urinary tract should be well hydrated, but not overly so, and have a partly filled, but not distended bladder.
  Conversely, dehydration may prevent the formation of hydronephrosis, thereby masking obstruction.
  Incorrectly ruling out a renal stone in the absence of hydronephrosis. Small stones may not cause hydronephrosis.
  Incorrectly ruling out ureterolithiasis, if no stone is visible. Mistaking renal cysts for hydronephrosis. The two can be distinguished with systematic real-time scanning through the entire kidney.
  Mistaking renal pyramids, which can be prominent in pediatric patients, for hydronephrosis. Their location is outside the renal sinus in contrast to hydronephrosis, which always occurs inside the renal sinus.
  Failure to identify unilateral absence of a kidney or horseshoe kidney. Both these conditions have important implications for the patient, and may affect acute management. Further diagnostic testing is likely to be indicated in such circumstances.
  Failure to visualize the entire kidney bilaterally and to evaluate the bladder

## Ultrasound-guided procedures

A unified reporting/quality assurance sheet for all procedures is presented at the end of the section.

### Ultrasound guidance for arthrocentesis

#### Evidence

1. Summary/brief explanation of indications (hip and knee)
  Arthrocentesis, also known as joint aspiration, is most commonly utilized to differentiate septic joint fluid from other types of joint effusions.
  In children who present with pain, limping or the inability to bear weight, ultrasound can assist arthrocentesis and result in more rapid diagnosis and treatment.
  Occasionally arthrocentesis may be done for therapeutic indications to relieve pain, either with medication injection or fluid removal.
  Dynamic ultrasound guidance uses real-time imaging to aid the procedure, with direct visualization of the needle into the joint space.
  Static technique is used to identify the location of the effusion relative to the skin surface and to mark an optimal location for needle insertion. The actual procedure is performed without ultrasound.
2. Relevant adult-specific literature
  Multiple studies demonstrate emergency physician success in using ultrasound to diagnose and aspirate hip effusions [213, 347–349].
  POCUS leads to greater fluid aspiration in knee arthrocentesis [350] and is better at identifying knee effusion than clinical examination alone [351].
3. Relevant pediatric-specific literature
  There are case reports of POCUS-guided arthrocentesis in pediatric patients [211, 241, 352].
4. Outstanding questions to be answered/voids in the literature to date
  POCUS-guided arthrocentesis in pediatric patients requires further investigation, specifically, the evaluation of success rates compared with landmark approaches, as well as evidence-based guidelines for training.
  Static versus dynamic technique is currently utilized based on operator preference.

#### Curriculum objectives (hip and knee)

1. Describe the* indications* for POCUS for arthrocentesis
  POCUS-guided arthrocentesis may be used for diagnostic or therapeutic aspiration of a joint effusion.
2. Describe the* limitations* of POCUS for arthrocentesis.
  Limitations include the following:
  The examination and procedure are dependent on both operator expertise and patient size/cooperation.
3. Describe the* relevant anatomy* to be identified during POCUS for arthrocentesis
  Hip:
    During imaging of the joint, the femoral head, femoral neck, acetabulum and iliopsoas muscle should be identified [353].
    Joint fluid will appear hypoechoic or anechoic in the anterior synovial space.
    The location of the femoral vessels should be identified sonographically prior to arthrocentesis.
    For those with experience in its use, the addition of color Doppler allows the provider to locate and avoid the femoral vessels during needle insertion [354].
  Knee:
    A joint effusion is detected with distension of the suprapatellar recess. Hypoechoic or anechoic fluid is within this recess [355].
    The effusion will appear as a hypoechoic fluid collection separated from the brightly echogenic femoral cortex by a thin layer of hyperechoic pre-femoral fat.
4. Recognize specific* pitfalls* involved in POCUS for arthrocentesis
  Incorrectly ruling out a septic joint due to lack of effusion, as infection may be present in the “normal” amount of joint fluid or only in the synovial tissue.
  Artifact induced false-positive effusions due to anisotropy of the muscles of the hip joint and lack of provider expertise [356].
  Misdiagnosing hypoechoic articular cartilage as an effusion, especially in hips of infants [357].
  Misdiagnosing a prepatellar bursitis as a joint effusion.
  Failure to observe the usual precautions and recommendations applicable to arthrocentesis whether performed with or without ultrasound guidance.

*Discussion of musculoskeletal ultrasound can be found in the diagnostic indications section.

### Ultrasound guidance for arterial catheter placement

#### Evidence

1. Summary/brief explanation of indications
  An arterial catheter is typically placed in critically ill or injured patients who require continuous hemodynamic monitoring and/or frequent laboratory testing.
  The radial and femoral arteries are frequently chosen for cannulation.
  The smaller size of these arteries in infants and children makes performing the procedure blindly more difficult.
  Ultrasound guidance may facilitate successful placement of arterial catheters.
2. Relevant adult-specific literature
  A randomized trial of 60 patients compared palpation to POCUS assistance for arterial cannulation. The ultrasound group demonstrated a shorter time to placement [107 vs. 314 s, (*p* = 0.004)], fewer attempts [1.2 vs. 2.2 (*p* = 0.001)], and fewer sites required for successful placement [1.1 vs. 1.6 (*p* = 0.001)] [358].
3. Relevant pediatric-specific literature
  A randomized trial of ultrasound-guided radial artery cannulation by anesthesiology trainees demonstrated higher overall as well as first attempt success rates, shorter time to identifying the vessel, and a smaller proportion of hematomas in the ultrasound group [359].
  A randomized trial of ultrasound-guided radial artery cannulation by pediatric anesthesiologists revealed no statistically significant difference in successful cannulation on first attempt, time to cannulation, or total number of attempts [360].
4. Outstanding questions to be answered/voids in the literature to date
  Trials are needed in pediatric patients comparing ultrasound assistance/guidance to palpation for arterial cannulation.
  Studies are needed to assess how many ultrasound-guided arterial catheters are needed to become proficient.
  It is unknown to what extent training and/or proficiency in ultrasound-guided venous access translates into that for ultrasound-guided arterial catheter placement.
  With newer, higher frequency, technology, ultrasound-guided arterial cannulation may prove more feasible.

#### Curriculum objectives

1. Describe the* indications* for ultrasound-assisted arterial catheter placement
  Ultrasound-assisted arterial catheter placement is indicated when an arterial catheter is needed for hemodynamic or blood gas monitoring in critically ill patients.
2. Describe the* limitations* for ultrasound-assisted arterial catheter placement
  User familiarity and experience with the procedure both with and without ultrasound can determine success of the procedure.
  The small size of the artery and depth (too shallow or too deep) can also hinder cannulation.
  Patient characteristics like movement, blood pressure, volume status, and body habitus will also pose a challenge for placement.
3. Describe the* relevant anatomy* to be identified while performing ultrasound-assisted arterial catheter placement
  The most common sites for arterial catheter placement in pediatrics are the radial and femoral arteries.
  A vessel can be visualized in either the short-axis (cross sectional) or the long-axis plane, along the length of the vessel.
  The radial artery originates from the brachial artery medial to the biceps tendon and continues to the styloid process of the radius. Up to 30 % of individuals have variants in the course of the radial artery. The portion of the radial artery in the distal forearm has less anatomical variation making it the preferable site for cannulation [361].
  The most easily accessible portion of the femoral artery is in the femoral triangle, made up of the inguinal ligament superiorly, the Sartorius muscle laterally and medially by the adductor longus muscle.
  The relationship between the femoral vein (medial to artery) and femoral nerve (lateral to artery) should be noted in an effort to minimize accidental cannulation of or injury to adjacent structures [362].
  If the operator is proficient in the use of Doppler, this modality can be used to distinguish the artery from veins and nerves.
4. Recognize specific* pitfalls* involved in performing ultrasound-assisted arterial catheter placement
  Small vessels like the radial artery may be compressed and, therefore, difficult to visualize with minimal pressure from the ultrasound transducer.
  Failure to confirm that the vessel is actually an artery, not a nerve or a vein. For those proficient in the use of Doppler, this modality can be used to distinguish the artery from veins and nerves.
  As with other procedures in pediatric patients, small patient size, and lack of patient cooperation may present additional challenges to successful cannulation of the artery.
  There may be significant overlap of the femoral vein and artery in the femoral triangle. Changes in leg position may reduce this overlap, but this will require immobilizing the patient in the optimal position throughout the procedure [362, 363].
  In the short-axis view, the operator must follow the needle tip at all times to avoid damage to the posterior wall of the vessel or other structures.
  In the long-axis view, the length of the vessel and entire needle should be visualized. This may not be possible if the vessel is not straight. It may also require greater operator skill since the transducer, needle, and vessel must be visualized in the same plane throughout the procedure. It may also require more advanced skill to distinguish an adjacent artery and vein in the longitudinal plane.
  Failure to observe the usual precautions and recommendations applicable to arterial catheter placement whether performed with or without ultrasound guidance.

### Ultrasound guidance for incision and drainage of soft tissue abscesses

#### Evidence

1. Summary/brief explanation of indications
  After ultrasound diagnosis of an abscess, ultrasound guidance can be used to facilitate surgical drainage of an abscess. Dynamic and static techniques can be used. In both cases, ultrasound is used to determine the size, depth, and location of the abscess, and to identify surrounding structures (e.g., nerves, vessels), which need to be avoided
    Dynamic ultrasound guidance uses real-time imaging with direct visualization of the needle or scalpel passing into the abscess.
    Using the static technique, an optimal location for incision or needle insertion is determined by ultrasound, and the skin is marked. The actual procedure is then performed without ultrasound.
2. Relevant adult-specific literature
  POCUS evaluation may identify surrounding vascular structures, and avoid potentially serious complications of incision and drainage [364].
  POCUS-guided needle aspiration has been shown to be insufficient in treating abscesses, when compared with incision and drainage [365].
3. Relevant pediatric-specific literature
  To date, there are no studies in pediatric patients specifically evaluating ultrasound-guided incision and drainage.
4. Outstanding questions to be answered/voids in the literature to date
  It is unknown whether ultrasound-guided drainage in pediatric patients is more effective and leads to improved outcomes compared with drainage without the use of ultrasound guidance.
  To date, there are no ultrasound studies on size and volume parameters for abscess drainage. The presence of a fluid collection on ultrasound does not necessarily indicate that drainage is needed.

#### Curriculum objectives

1. Describe the* indications* for ultrasound-guided incision and drainage
  Ultrasound may assist in determining the extent of surgical drainage by revealing the depth and boundaries of abscesses (e.g., in the distinction between perianal versus perirectal abscess).
  Ultrasound can identify the presence of infection involving deeper structures (e.g., tenosynovitis) in infections that appear to be limited to the superficial soft tissues.
  Ultrasound may identify the presence of foreign bodies in soft tissue infections.
  Ultrasound-guided needle aspiration may be useful in those areas where the lesion is in close proximity to vascular and nerve structures.
  Ultrasound can help to guide surgical management (needle aspiration versus incision and drainage) in cosmetically sensitive locations (e.g., the face).
  Ultrasound can be used to ensure that a lesion has been completely drained.
2. Describe the* limitations* of ultrasound-guided incision and drainage
  At this time, there are no known limitations or contraindications to sonographic evaluation of soft tissue abscesses prior to surgical drainage.
  The current literature demonstrates the inferiority of needle aspiration under ultrasound guidance compared to incision and drainage in terms of clinical resolution [365].
3. Describe the* relevant anatomy* to be identified during ultrasound-guided incision and drainage
  An abscess is identified as a heterogeneous hypoechoic, occasionally isoechoic, irregularly shaped lesion with posterior acoustic enhancement. Gas or foreign bodies may also be seen.
  Vessels and nerves in the region of the abscess should be positively identified prior to surgical drainage.
  The superficial fascia of underlying musculoskeletal structures should be identified to exclude involvement of deeper structures. Color flow may be useful to identify vessels and lymph nodes.
4. Recognize specific* pitfalls* involved in ultrasound-guided incision and drainage
  Mistaking a vessel, hematoma, or lymph node for an abscess.
  Failure to recognize the full extent or depth of an abscess. This may be particularly challenging with perianal or gluteal infections.
  Failure to recognize that an infection is arising from structures below the dermis.
  Failure to observe the usual precautions and recommendations applicable to incision and drainage whether performed with or without ultrasound guidance.

*Discussion of skin and soft tissue ultrasound can be found in the diagnostic indications section.

### Ultrasound guidance for lumbar puncture

#### Evidence

1. Summary/brief explanation of indication
  The lumbar puncture (LP) is routinely performed in the emergency department for febrile neonates, children with a clinical suspicion for meningitis or encephalitis, and in patients with suspected or diagnosed idiopathic intracranial hypertension.
  Ultrasound can identify the spinous processes, interspinous spaces, and guide needle placement via static or dynamic imaging.
  Ultrasound may be particularly useful in neonates who have small interspinous spaces as well as obese patients where palpation of anatomical landmarks is difficult.
  In neonates, the spine is largely cartilaginous allowing for visualization of the spinal canal including the level of conus medullaris. Ossification of the vertebra occurs around 1 year of age.
  Ultrasound assistance (‘static technique’) is the use of ultrasound prior to lumbar puncture to determine an optimal location for needle insertion. The procedure is then performed without ultrasound.
  Dynamic ultrasound-guidance provides direct visualization of the passage of the needle from the skin into the spinal canal.
2. Relevant adult-specific literature
  Tuffier’s line (the line between the superior portion of posterior iliac crests) is commonly used to estimate the L4 vertebra. In a study by Pysyk, ultrasound showed that Tuffier’s line is not a reliable method to consistently identify a particular vertebral space [366].
  In adult patients, POCUS has been shown to be highly effective in identifying bony landmarks for LP, even in those who are obese [367, 368].
  In one study, the success rate of ultrasound-assisted LP was found to be 92.3 %, regardless of the subjects’ body mass index (BMI) [369].
  In a randomized controlled trial comparing POCUS with the traditional landmark approach, POCUS had a success rate of 95.8 % and was more likely to be successful (RR 1.32, 95 % CI 1.01–1.72). In obese patients, with BMI ≥ 30, there were fewer failed attempts in the POCUS group (RR 2.33, 95 % CI 0.99–5.49) [370].
  Another randomized controlled trial of 80 patients found that ultrasound-assisted LP was associated with decreased procedure time, number of attempts, traumatic tap rate, and pain score during the procedure. Such benefits seem to be more prominent in those with a higher BMI [371].
3. Relevant pediatric-specific literature
  Pediatric studies have shown that POCUS can be used to measure the interspinous space and to determine how the measurement changes in different positions [372, 373]. The interspinous space is maximized in a seated position with hip flexion. In the decubitus position, neck flexion does not change the interspinous space and, thus, the neck should not be flexed for the procedure [372].
  Another pediatric study used POCUS to assess the angle of needle placement [374]. It demonstrated that the angle for children under 12 months was significantly less (on average 50° to the skin, directed cephalad) than that for patients above this age (on average 60°).
  In neonates, ultrasound can be used to identify the level of conus medullaris [375, 376] as well as evaluate reasons for failed LP attempts [377].
  In 2014, a feasibility study conducted on 19 patients found that using POCUS was associated with higher confidence score in selecting the insertion site for the LP needle compared to the traditional landmark approach [378].
  Preliminary data (*n* = 26) from a randomized controlled trial of infants 0-12 months found no significant difference between the groups (POCUS vs. landmark approach) in terms of LP success rate, traumatic LP rate (RBC > 10,000/mm^3^ on CSF analysis), number of LP attempts, or total LP duration [379].
4. Outstanding questions to be answered/voids in the literature to date
  To date, there are no large randomized controlled trials investigating the effect of POCUS on success rates, complications, costs, patient flow, or patient satisfaction related to LPs performed in the ED.
  Investigations are needed to determine the comparative effectiveness of static vs. dynamic techniques of ultrasound guidance.

#### Curriculum objectives

1. Describe the* indications* for POCUS-assisted LP
  Patients in whom bony landmarks of the spinous processes are difficult to palpate.
  Patients in whom the interspinous spaces are small and difficult to identify.
  Patients in whom LP has failed using the landmark approach.
2. Describe the* limitations* of POCUS-assisted LP
  Inability to visualize the anatomy sonographically for any reason.
  After vertebral ossification, ultrasound is limited to visualization of the spinous processes and interspinous spaces.
  Movement of the patient prior to needle insertion after marking by ultrasound using the static technique may alter the location of underlying structures, especially in young infants.
  Success is dependent on the operator and the level of experience with ultrasound as well as with the LP procedure.
3. Describe the* relevant anatomy* for POCUS-assisted LP
  It is most important to identify the spinous processes and the interspinous spaces.
  The spinous processes will be the most superficial bony prominence that is palpated clinically and visualized sonographically.
  In neonates, the spine is mostly cartilaginous allowing for visualization of the anatomy from the spine to the spinal canal. In this case, ultrasound can identify the skin, spinous process, interspinous space, ligaments, epidural space, dura/arachnoid, and subarachnoid space. Within the subarachnoid space, ultrasound can visualize the spinal canal, conus medullaris, cauda equina, as well as, the filum terminale.
4. Recognize specific* pitfalls* involved POCUS-assisted LP
  Misidentifying the transverse process as the spinous process as a result of the transducer being misplaced laterally and not in the midline.
  Mistaking the spinal cord as cerebrospinal fluid in the subarachnoid space.
  Failure to observe the usual precautions and recommendations applicable to lumbar puncture whether performed with or without ultrasound guidance.

### Ultrasound guidance for paracentesis

#### Evidence

1. Summary/indications
  Ascites may be present in patients with nephrotic syndrome, malnutrition, malignancy, congestive heart failure, pancreatitis, bacterial peritonitis, non-bacterial causes of peritoneal inflammation, tuberculosis or dengue fever. Other causes of abdominal fluid collections include biliary and urinary tract injury, which may be iatrogenic in etiology.
  POCUS can identify small amounts of free fluid in the abdominal cavity, and can be useful in its diagnosis. Symptoms of abdominal fluid collections range from asymptomatic to pain, distension and respiratory distress.
  Using ultrasound to localize intra-abdominal fluid and to perform paracentesis involves direct visualization of fluid, as well as structures to be avoided during the procedure including bowel, the urinary bladder, and the inferior epigastric vessels.
    Dynamic ultrasound guidance uses real-time imaging to aid paracentesis with direct visualization of the needle trajectory.
    Static technique is used to identify anatomic landmarks and the extent of the peritoneal fluid, and to determine an optimal location for needle insertion. The actual procedure is performed without ultrasound.
2. Relevant adult-specific literature
  In a prospective randomized study, Nazeer et al. showed that ultrasonographic guidance not only improves the rate of successful paracentesis by emergency physicians, but also helps determine the need for the procedure, thus reducing unnecessary interventions and patient discomfort [380].
  Patel et al. performed a retrospective analysis comparing blind and ultrasound-assisted paracentesis. Adverse events (post-paracentesis infection, hematoma, seroma) were lower (1.4 vs 4.7 % *p* = 0.01) as well as hospital costs in the ultrasound-assisted group [381]. An additional study also showed decreased bleeding complications and hospitalization cost [382].
  One case series suggested that ultrasound-guided, emergent paracentesis in the management of unstable, hypotensive patients assisted in the characterization of the fluid, thereby guiding patient management [383].
3. Relevant pediatric-specific literature
  There have been no studies to date evaluating POCUS for paracentesis in pediatric patients.
4. Outstanding questions to be answered/voids in the literature to date
  There are currently no published investigations of the relative benefits and risks of ultrasound-guided paracentesis compared to traditional technique with respect to complication rates, patient satisfaction, pain, or other outcomes. However, the infrequent need for paracentesis in pediatric patients would make design of such studies difficult. Under such circumstances, it may be justifiable to generalize the favorable risk:benefit profile demonstrated in adult patients to the pediatric setting.
  It is unclear how much training and how many procedures are required to attain competency.

#### Curriculum objectives

1. Describe the* indications* for POCUS for paracentesis
  POCUS for paracentesis should be used in those patients with suspected free intra-abdominal fluid in order to diagnose ascites, assist with fluid sampling for diagnosis, and for symptomatic relief.
  Once intraabdominal fluid is identified, POCUS is indicated to identify the largest pocket of fluid and intra-abdominal structures that need to be avoided during the procedure. Additionally, with color Doppler, POCUS may assist in the identification and location of the inferior epigastric vessels.
2. Describe the* limitations* of POCUS for paracentesis
  As with all ultrasound procedures, use is operator dependent and requires training.
  Ultrasound usually cannot differentiate between types of free fluid.
  Even with optimal technique, bowel can move within the abdomen, and therefore, perforation is still possible particularly when the static technique is used.
3. Describe the* relevant anatomy* to be identified with POCUS for paracentesis
  The first step is to identify pockets of free fluid. The technique is similar to the one used during the FAST examination. It consists of examination of bilateral upper and lower quadrants, the hepatorenal and splenorenal spaces, and the pelvis. The peritoneal lining is hyperechoic and pockets of ascites are anechoic or hypoechoic.
  When free fluid is identified, the area should be interrogated to assure that there is a direct unobstructed line from the skin to the target fluid collection. Intra-abdominal organs and vessels need to be identified to avoid them during puncture. The inferior epigastric vessels arise from the external iliac vessels immediately superior to the femoral canal and course superiorly along the approximate line of the lateral margin of the rectus abdominis muscle. They are located immediately deep to the abdominal muscle layers and superficial to the peritoneum. These should be identified and their trajectory and course drawn on the patient’s skin, designating an area to avoid. Color Doppler should be used to identify the inferior epigastric vessels, with the threshold set low.
  It is important to recognize that a full bladder might simulate free fluid. If a Foley catheter is not placed prior to performing the procedure, the sonologist should make positive identification of the dome of the bladder prior to the procedure.
  Once the fluid collection and surrounding structures are identified, it is important to maintain the patient’s position, in order to avoid shifts in fluid and structures.
4. Recognize the specific* pitfalls* involved in POCUS for paracentesis
  Movement of the patient after fluid mapping may lead to a shift in the fluid or bowel, especially if the static technique is used.
  Failure to visualize the largest pocket of fluid.
  Insertion of the needle in close proximity to bowel.
  Mistaking the bladder or cystic masses for ascites.
  Failure to observe the usual precautions and recommendations applicable to paracentesis whether performed with or without ultrasound guidance.

### Ultrasound guidance for pericardiocentesis

#### Evidence

1. Summary/brief explanation of indications
  Pericardiocentesis is the aspiration of fluid, blood, or pus, from the space between the visceral and parietal pericardium.
  Use of ultrasound guidance to perform pericardiocentesis has been described since 1979, and has fewer complications than performing the procedure blindly [384].
  Indications for a therapeutic pericardiocentesis are impending or current cardiac tamponade. In children, this usually occurs post-cardiac surgery or from traumatic injury.
    Dynamic ultrasound guidance uses real-time imaging to aid the procedure with direct visualization of the needle into the pericardial space.
    Static technique is used to identify the location of the effusion relative to the chest wall and to mark an optimal location for needle insertion. The actual procedure is performed without ultrasound.
2. Relevant adult-specific literature
  There are several case reports which describe ultrasound-assisted pericardiocentesis [385–387].
  An in-plane approach has been described [388].
3. Relevant pediatric-specific literature
  To date, there are no studies evaluating pediatric emergency physician-performed POCUS for pericardiocentesis.
4. Outstanding questions to be answered/voids in the literature to date
  Pericardial tamponade is a very rare condition, particularly in pediatric patients. This is likely the reason for the paucity of literature describing ultrasound-guided pericardiocentesis.
  Given the infrequency with which it is performed, it is not clear what method is best (e.g., simulation, animal models) for achieving competence with the procedure.

#### Curriculum objectives

1. Describe the *indications* for ultrasound-assisted pericardiocentesis
  The only indication for emergent ultrasound-assisted pericardiocentesis is life-threatening cardiac tamponade.
  The non-emergent indication is the need for sampling pericardial fluid for diagnostic purposes.
2. Describe the *limitations* of ultrasound-assisted pericardiocentesis
  Success is largely dependent on operator comfort and experience with the procedure.
  Transducer placement and site of needle entry may be limited by the small size of the child.
3. Describe the *relevant anatomy* to be identified during ultrasound-assisted pericardiocentesis
  The heart, specifically the right and left ventricles, liver, pericardium and pericardial space are all structures that must be identified prior to beginning the procedure.
  A pericardial effusion will appear as an anechoic (black) space surrounding the heart contained by the bright echogenic pericardium.
  Tamponade is demonstrated by poor filling and/or diastolic collapse of the right side of the heart (right atria or right ventricle).
  Tamponade can occur with as little as 50 ml of fluid, if the fluid rapidly accumulates such as with blood from trauma. Tamponade can also occur with considerably more fluid if the accumulation is more gradual, such as in rheumatologic or oncologic conditions [389].
  Images can be obtained in the parasternal long, apical, or subxiphoid view, in order to determine best placement of the needle based on where the effusion is closest to the transducer.
4. Recognize specific *pitfalls* involved in ultrasound-assisted pericardiocentesis
  As with other ultrasound-guided procedures, the procedure may be complicated by patient characteristics, including body habitus, and movement during the procedure.
  Failure to determine the appropriate needle entry site and trajectory while avoiding vital structures and accessing the largest fluid accumulation closest to body surface [384].
  Failing to recognize and avoid the internal mammary artery (3–5 cm lateral to the lower sternal boarder) and the neurovascular bundles that run below each rib [389].
  Laceration of myocardial tissue or vessels due to side-to-side needle manipulation during entry through the heart.
  Failure to observe the usual precautions and recommendations applicable to pericardiocentesis whether performed with or without ultrasound guidance.

*Discussion of cardiac ultrasound can be found in the diagnostic indications section.

### Ultrasound guidance for regional anesthesia

#### Evidence

1. Summary/brief explanation of indications
  Use of ultrasound to guide regional anesthesia in patients has greatly improved the ability to deliver safe and effective analgesia.
  The majority of the literature surrounds anesthesiologist-performed ultrasound for this indication.
    Dynamic ultrasound guidance uses real-time imaging to perform the nerve blockade, with direct visualization of the needle path to the nerve bundle.
    Static technique is used to identify the location of the nerve and surrounding vessels to avoid, and to mark an optimal location for needle insertion. The actual procedure is performed without ultrasound.
2. Relevant adult literature
  A small study of emergency department (ED) patients demonstrated that emergency physicians could easily learn ultrasound-guided nerve blockade, and perform the procedure effectively and without complications [390].
  In another small study of patients assigned to either procedural sedation or ultrasound-guided supraclavicular brachial plexus nerve blockade, the mean ED length of stay was nearly 3 h shorter in the nerve blockade group [391].
3. Relevant pediatric literature
  Anesthesiologist-performed ultrasound allows for visualization of peripheral nerves and their surrounding anatomy in real time, thereby enabling reliable drug delivery to the target nerve while avoiding mechanical nerve injury, local and systemic drug toxicity, or injection into adjacent structures in pediatric patients [392–394].
  A retrospective study of pediatric emergency physician-performed ultrasound-guided femoral nerve blocks showed that patients had longer duration of analgesia, required fewer doses of analgesic medications, and needed fewer nursing interventions than those receiving analgesic medication alone [395].
  A feasibility study of pediatric emergency physician-performed ultrasound-guided forearm nerve blocks showed effective analgesia, minimized iatrogenic risk and procedure time ideal for emergency department workflow [396].
4. Outstanding questions to be answered/voids in the literature to date
  There are limited data to compare pediatric emergency physician-performed ultrasound-guided regional anesthesia to traditional methods of pain control or anesthesia.
  Further study may include novel techniques, anesthesia agents, and target nerves that are pediatric-specific.
  Scientific information is needed to determine the costs and benefits of ultrasound-guided regional anesthesia with respect to success rates, complications, costs, ED length of stay, and patient satisfaction.

#### Curriculum objectives

1. Describe the* indications* for ultrasound-guided regional anesthesia
  The indication for ultrasound-guided regional anesthesia includes any pain control scenario in which analgesia in the distribution of a peripheral nerve or nerves is desired for pain control or anesthesia. Examples include: fractures, dislocations, burns, abscess incision and drainage, foreign body removal, and laceration repair.
2. Describe the* limitations* of ultrasound-guided regional anesthesia
  An uncooperative patient can limit successful nerve blockade. Patients may benefit from topical anesthesia and anxiolytic adjuncts to minimize pain with needle entry and patient anxiety during the procedure. Limiting patient movement using age-appropriate restraints may also be necessary.
  The inability to visualize a target nerve or deliver the anesthetic agent to the perineural area due to patient anatomy should prompt alternative pain control measures.
3. Define the* relevant anatomy* associated with commonly used nerve blocks including: interscalene, supraclavicular, forearm, intercostal, femoral, and popliteal nerve blocks
  Depending on the size of the patient’s limb and the footprint of the transducer, the target nerve can be visualized in cross section or longitudinally. Where both views are available, many sonologists prefer to view the nerve in cross section, allowing for demonstration of anesthetic solution surrounding the nerve.
  Regardless of the scanning plane with respect to the nerve, most sonologists prefer to introduce the needle in the plane of the ultrasound transducer. The in-plane approach allows for visualization of the entire length of the needle, and with injection, the anesthetic should be seen surrounding the target nerve, creating a halo.
  Interscalene block—The brachial plexus trunks should be identified, as well as the middle and anterior scalene muscles and the sternocleidomastoid muscle.
  Supraclavicular block—The brachial plexus divisions should be identified as well as the subclavian artery, the first rib and the pleural line.
  Forearm nerve block—The radial, median and ulnar nerves should be identified as well as the radial and ulnar arteries.
  Intercostal nerve block—The intercostal space should be identified at the intended level of the block as well as the inferior margin of the rib and the pleural line.
  Femoral nerve block—The femoral nerve should be identified as well as the femoral artery, fascia lata and fascia iliacus.
  Popliteal nerve block—The popliteal, tibial and common peroneal nerves, and their points of bifurcation should be identified as well as the popliteal artery and vein.
4. Recognize the* pitfalls* associated with ultrasound-guided regional anesthesia
  Failure to maintain continuous visualization of the needle tip in-plane during entry and injection.
  Complications to be avoided include: neuronal injury by direct mechanical trauma, vascular puncture and vascular injection of anesthesia agent.
  Pneumothorax is a rare potential complication for regional anesthesia of the brachial plexus and intercostal nerves.
  Failure to observe the usual precautions and recommendations applicable to regional anesthesia whether performed with or without ultrasound guidance.

### Ultrasound guidance for suprapubic bladder aspiration

#### Evidence

1. Summary/brief explanation of indications
  In addition to assessing for adequate bladder volume prior to catheterization, POCUS may be used to assist in performing suprapubic bladder aspiration (SPA).
    Dynamic ultrasound guidance uses real-time imaging to aid SPA with direct visualization of the needle trajectory.
    Static technique is used to identify the size and location of the bladder and to mark an optimal location for needle insertion. The actual procedure is performed without ultrasound.
2. Relevant adult-specific literature
  There are few studies in adult patients, since suprapubic aspiration is uncommonly performed in adult patients.
3. Relevant pediatric-specific literature
  The failure rate of SPA without ultrasound guidance is reported as 8 % or higher [397].
  When ultrasound was performed prior to SPA to evaluate for the presence of sufficient urine in the bladder, first attempt success was shown to be 100 %, when compared with 36 % without prior ultrasound evaluation [398].
  Ultrasound-guided SPA has been shown to be more successful compared to blind SPA [399, 400].
  The use of the bladder scan which is a portable ultrasound device prior to suprapubic aspiration has been shown to have success rates of 53 %, which is lower than reported success rates of real-time ultrasound [401].
4. Outstanding questions to be answered/voids in the literature to date
  Given the frequency with which urethral catheterization is performed today, in lieu of SPA, it is not clear what method is best (e.g., simulation, animal models) for training and demonstrating competence with the procedure.

#### Curriculum objectives

1. Describe the* indications* for POCUS for suprapubic aspiration
  Indications for performing bladder ultrasound include patients in whom a sterile urine specimen is needed and who are unable to voluntarily provide a specimen (i.e., children less than 2 years of age, or those who have limited mobility), or in whom urethral catheterization is unsuccessful.
2. Describe the* limitations* of POCUS for suprapubic aspiration or urethral catheterization.
  Success is dependent on operator comfort and their level of experience with suprapubic aspiration, which is uncommon in many settings.
3. Describe the* relevant anatomy* to be identified in the POCUS examination for suprapubic aspiration or urethral catheterization
  The bladder is recognized as an anechoic structure with a surrounding thin echogenic line, indicating its wall.
  Imaging in the sagittal plane and introducing the needle in the plane of the ultrasound beam are preferred for ultrasound guidance, to visualize the entire length of the needle.
4. Recognize specific* pitfalls* involved in POCUS for suprapubic aspiration or urethral catheterization
  An empty (i.e., collapsed) bladder may not be identified by ultrasound examination.
  Other fluid-filled structures, e.g., loops of bowel with ileus, must be distinguished from bladder.
  Not following the full trajectory of the needle may result in penetration of other structures (e.g., bowel).
  Failure to observe the usual precautions and recommendations applicable to suprapubic or urethral catheterization whether performed with or without ultrasound guidance.

*Discussion of bladder ultrasound can be found in the diagnostic indications section.

### Ultrasound guidance for thoracentesis

#### Evidence

1. Summary/indications
  Lung ultrasound allows timely recognition, characterization and precise localization of pleural effusions. Ultrasound allows visualization of structures to be avoided during the procedure such as the diaphragm and lung parenchyma.
  Ultrasound-guided thoracentesis has been shown to reduce iatrogenic complications and to increase the success of thoracentesis.
    Dynamic ultrasound guidance uses real-time imaging to aid thoracentesis with direct visualization of the needle trajectory.
    Static technique is used to identify anatomic landmarks and the extent of the pleural effusion, and to mark an optimal location for needle insertion. The procedure is then performed without real-time ultrasound guidance.
2. Relevant adult-specific literature
  Ultrasound-guided thoracentesis reduces rates of pneumothorax [382, 402–408], increases rates of success [382, 402, 404–408] and reduces complications such as the need for subsequent tube thoracostomy [403, 404, 406–408].
  Additional studies suggest that ultrasound-guided thoracentesis is associated with lower hospital length of stay, costs, and a lower incidence of pneumothoraces [382, 407].
3. Relevant pediatric-specific literature
  There have been no studies to date evaluating POCUS for thoracentesis in pediatric patients.
4. Outstanding questions to be answered/voids in the literature to date
  As no randomized controlled trials of POCUS for thoracentesis in pediatric patients exist, it is unknown whether there is a benefit in procedure duration, complication rate, patient/parent satisfaction, pain, or outcome. The favorable risk:benefit profile demonstrated in some studies of adult patients may be translated to the pediatric setting.

#### Curriculum objectives

1. Describe the* indications* for POCUS for thoracentesis
  POCUS for thoracentesis should be used whenever a thoracentesis is to be performed, especially when a difficult procedure is anticipated.
2. Describe the* limitations* of POCUS for thoracentesis
  Thoracentesis is an invasive procedure with potential complications such as pneumothorax and injury of solid organs or the diaphragm.
3. Describe the* relevant anatomy* to be identified in the ultrasound for thoracentesis
  The optimal site for drainage of a non-loculated pleural effusion is usually on the posterior axillary line above the diaphragm. POCUS can confirm this or prompt the choice of an alternative location. The hemithorax of interest should be scanned from the inferior border of the scapula to the upper lumbar region and the costophrenic sulcus should be evaluated from the paravertebral region posteriorly to the parasternal region anteriorly.
  Structures such as the diaphragm, subdiaphragmatic organs (spleen and liver) and thoracic organs (lung, heart and ribs) should be identified before the procedure. Ideally, a rib-space above and below the site of thoracentesis and a pocket at least 15 mm deep should be identifiable throughout the respiratory cycle at the site selected for thoracentesis.
  In loculated effusions, a space, usually anechoic, is identified in the costophrenic sulcus between the parietal and visceral pleura. Fluid moves with patient positioning and will accumulate in the most dependent areas of the hemithorax; thus, the upright position is optimal.
  The presence of septations and/or fibrinous strands usually suggests an exudate and/or a loculated collection. A loculated effusion may appear as multiple pockets of fluid separated by septations. It will not be mobile and, therefore, may not be dependent.
4. Recognize the specific* pitfalls* involved in POCUS for thoracentesis
  Free fluid in the abdominal cavity could be misidentified as a pleural effusion if the diaphragm is not identified, especially when there is an elevated hemidiaphragm.
  The patient should not move after the effusion is mapped, as fluid can shift.
  Not visualizing the lung and diaphragm through all phases of the respiratory cycle may result in organ injury.
  Recommendations regarding safe volumes for evacuation during a single thoracentesis are beyond the scope of this document.
  Failure to observe the usual precautions and recommendations applicable to thoracentesis whether performed with or without ultrasound guidance.

*Discussion of lung ultrasound can be found in the diagnostic indications section.

### Ultrasound guidance for vascular access

#### Evidence

1. Summary/brief explanation of indications
  Venous access can be a life-saving procedure for many children who present to the emergency department.
  Smaller children, especially those with extensive medical histories, and volume-depleted patients pose particular difficulties with venous access.
  Ultrasound guidance may facilitate peripheral intravenous (IV) access, thereby avoiding central catheter placement or intraosseous needle placement, which can be associated with complications.
  There are international recommendations regarding the use of ultrasound for central and peripheral venous access [409]/
    Dynamic ultrasound guidance uses real-time imaging with direct visualization of the needle passing into the vessel. Most of this information that follows relates to the dynamic technique.
    Using static ultrasound guidance, an optimal location for needle insertion is determined by ultrasound, and the skin is marked. The actual procedure is then performed without real-time ultrasound guidance. This technique is useful for practitioners who have not developed skill in dynamic ultrasound-guided vascular access.
2. Relevant adult-specific literature
*Central venous access* Ultrasound has been shown to facilitate central venous access in adult patients. When compared with the landmark method, ultrasound guidance was shown to have a lower overall and first attempt failure rate [410].
  Compared with traditional landmark-guided approaches, ultrasound guidance results in fewer complications, mean insertion attempts, and placement failures [411, 412].
  A randomized study of three different approaches to internal jugular venous cannulation evaluated the success of the landmark approach compared to dynamic and static ultrasound guidance. Dynamic guidance 54 times the odds of successful cannulation compared to the landmark approach and the static approach had three times the odds of success compared to the landmark approach [413].
  A 2015 Cochrane systematic review of 35 studies (31 of adult patients) of ultrasound guidance for internal jugular vein cannulation concluded that ultrasound offered “gains” in both safety and quality compared with an anatomical landmark technique [414]. The Cochrane review of 13 studies (11 of adult patients) of ultrasound guidance for femoral and subclavian cannulation concluded that there were only “small gains” in safety and quality compared with an anatomical landmark method [415].
  The Institute of Medicine recommends ultrasound guidance as standard of care in the placement of all central catheters to improve patient safety [416]. The use of ultrasound guidance for central venous cannulation is listed as one of the top 11 methods to improve patient safety by the Agency for Healthcare Research and Quality [417].
*Peripheral venous access* When compared with traditional cannulation techniques, ultrasound-guided peripheral intravenous catheter placement has greater success rates, fewer skin punctures, decreased time for intravenous catheter placement, and fewer complications [418, 419].
  The use of ultrasound-guided peripheral intravenous catheter placement is associated with decreased use of central venous catheter use [420].
*Intraosseous placement* One study in eight cadaveric legs demonstrated the ability of ultrasound using color power Doppler to accurately assess placement of intraosseous needles [421].
3. Relevant pediatric-specific literature
*Central venous access* Ultrasound has been show to facilitate central venous access in pediatric patients. In surgical patients, the overall success rate for ultrasound guidance was 91.5 % compared with 72.5 % in the landmark-guided group. However, in children less than 1 year of age and less than 10 kg, the success rates were not statistically different [422].
  In pediatric patients, surgeon-performed central venous access was more successful with ultrasound guidance than the landmark approach, with first attempt success in 65 % patients in the US group, compared with 45 % in the landmark group (*p* = 0.02); success was achieved within three attempts in 95 % of the ultrasound group vs. 74 % of the landmark group (*p* < 0.001) [423].
  When evaluating the femoral vessels with ultrasound, it was found that external landmarks were not always predictive of internal anatomy. The femoral vein may be completely or partially overlapped by the femoral artery in 12 % of patients. Therefore, visualization by ultrasound is recommended prior to femoral vein catheterization [424].
  Additional confirmation of proper central catheter placement in children involves flushing the central catheter with agitated saline solution [425].
*Peripheral venous access* One study of ultrasound for procedural-guidance revealed no change in overall success rates, but decreased time to placement, decreased number of attempts, and decreased number of needle redirections [426].
*Intraosseous placement* One case series of five critically ill patients describes the use of ultrasound with color Doppler to correctly identify proper and improperly positioned intraosseous needles [427].
4. Outstanding questions to be answered/voids in the literature to date
  Further investigation is needed in pediatric patients to address the overall success rate of ultrasound-guided peripheral IV access compared with blind insertion.
  It is unclear how much training and how many ultrasound-guided catheter placements should be required to become competent in the procedure.

#### Curriculum objectives

1. Describe the* indications* for ultrasound-guided vascular access
  Those patients with a history of difficult access, or who at the time of presentation have had failed attempts at access by traditional methods.
2. Describe the* limitations* of ultrasound guidance for vascular access.
  Limitations of vascular access ultrasound include the following:
  Success of ultrasound-guided vascular access is largely dependent on the operator and his/her level of experience.
  Regardless of whether ultrasound is used for vascular access, there are still some intrinsic difficulties with the veins themselves: valves, collapsing and rolling.
  Smaller diameter vessels, and deeper vessels are often more difficult to cannulate [428].
3. Describe the* relevant anatomy* to be identified when using ultrasound for vascular access
  Vessels should be “pre-scanned” in longitudinal and transverse planes to identify important surrounding structures, anatomic anomalies, and to determine the optimal site(s) for cannulation.
  The most common sites for central catheter placement include the femoral vein, internal jugular vein, and subclavian vein.
  Peripheral venous cannulations are commonly performed in the basilic, brachial, cephalic, and saphenous veins.
  Vessels may be cannulated in the short-axis (“out of plane”), or in the long-axis (“in plane”).
4. Recognize specific* pitfalls* involved in ultrasound-*guided vascular access*
  Procedural ultrasound may be complicated by patient characteristics, including large body habitus and movement during the procedure or after marking during a static procedure.
  When utilizing the short-axis technique, errors in placement of the needle or positioning of the transducer may lead to erroneous positioning of the needle in relation to the vessel.
  Using the short-axis technique, the most common error is failure to maintain constant localization of the needle tip. The needle tip should be guided in real time into the lumen of the vessel.
  The long-axis technique allows for visualization of the entire path of the needle, but is technically more challenging since the transducer, needle, and vessel need to remain continuously in the same plane.
  “Flashback” of blood into the needle chamber occurs as the bevel of the needle enters the vessel lumen. Advancing the catheter over the needle at this point will result in failure of cannulation. The needle and catheter should be advanced together for several millimeters after “flashback” in order for the catheter to enter the vessel.
  Veins must be distinguished from nerves and arteries with which they frequently travel. Nerves are non-compressible and will demonstrate internal echoes when the scanning plane is exactly perpendicular to the nerve.
  Arteries may compress and be difficult to distinguish from veins, resulting in arterial cannulation. This is especially common in younger children. The operator should look for arterial pulsations or Doppler color flow to further distinguish arteries from veins. It may be particularly difficult to distinguish an artery from a vein in a patient in shock.
  Failure to observe the usual precautions and recommendations applicable to regional anesthesia whether performed with or without ultrasound guidance
