# Erratum to: Pediatric emergency medicine point-of-care ultrasound: summary of the evidence

**DOI:** 10.1186/s13089-017-0058-z

**Published:** 2017-02-03

**Authors:** Jennifer R. Marin, Alyssa M. Abo, Alexander C. Arroyo, Stephanie J. Doniger, Jason W. Fischer, Rachel Rempell, Brandi Gary, James F. Holmes, David O. Kessler, Samuel H. F. Lam, Marla C. Levine, Jason A. Levy, Alice Murray, Lorraine Ng, Vicki E. Noble, Daniela Ramirez-Schrempp, David C. Riley, Turandot Saul, Vaishali Shah, Adam B. Sivitz, Ee Tein Tay, David Teng, Lindsey Chaudoin, James W. Tsung, Rebecca L. Vieira, Yaffa M. Vitberg, Resa E. Lewiss

**Affiliations:** 1 Children’s Hospital of Pittsburgh, 4401 Penn Ave, AOB Suite 2400, Pittsburgh, PA 15224 USA; 2 Children’s National Medical Center, Washington, DC USA; 3 Maimonides Medical Center, Brooklyn, NY USA; 4 New York Methodist Hospital, Brooklyn, NY USA; 5 Hospital for Sick Children, Toronto, ON Canada; 6 Boston Children’s Hospital, Boston, MA USA; 7 Queens Medical Center, Honolulu, HI USA; 8 University of California-Davis, Sacramento, CA USA; 9 Morgan Stanley Children’s Hospital, New York, NY USA; 10 University of California-San Diego, San Diego, CA USA; 11 Dell Children’s Medical Center, Austin, USA; 12 Boston Medical Center, Boston, MA USA; 13 Massachusetts General Hospital, Boston, MA USA; 14 Boston University Medical Center, Boston, NY USA; 15 Columbia University Medical Center, New York, NY USA; 16 St. Lukes-Roosevelt Hospital, New York, NY USA; 17 Montefiore Medical Center, Bronx, NY USA; 18 Newark Beth Israel Medical Center, Newark, NJ USA; 19 Mount Sinai Hospital, New York, NY USA; 20 Cohen Children’s Medical Center, New Hyde Park, USA; 21 Weill Cornell Medical Center, New York, NY USA; 22 University of Colorado, Aurora, CO USA

## Erratum to: Crit Ultrasound J (2016) 8:16 DOI 10.1186/s13089-016-0049-5

Since the publication of our article [1], it has come to our attention that we omitted to thank the American College of Emergency Physicians (ACEP) for their permission to use ACEP Standardized Reporting Guidelines (SRGs) in the development of the SRGs proposed in our article. The full citation for the ACEP SRGs is as follows: American College of Emergency Physicians. Emergency Ultrasound Standard Reporting Guidelines [information paper]. October 2011. https://www.acep.org/workarea/DownloadAsset.aspx?id=104378. Accessed November 22, 2016. The SRGs in our article should have carried the following credit line: © 2011–2016, American College of Emergency Physicians, Adapted with Permission.

## References

[1] Marin JR, Abo AM, Arroyo AC, Doniger SJ, Fischer JW, Rempell R (2016). Pediatric emergency medicine point-of-care ultrasound: summary of the evidence. Crit Ultrasound J.

